# The molecular architecture of *Lactobacillus* S-layer: Assembly and attachment to teichoic acids

**DOI:** 10.1073/pnas.2401686121

**Published:** 2024-06-05

**Authors:** Theo Sagmeister, Nina Gubensäk, Christoph Buhlheller, Christoph Grininger, Markus Eder, Anđela Ðordić, Claudia Millán, Ana Medina, Pedro Alejandro Sánchez Murcia, Francesca Berni, Ulla Hynönen, Djenana Vejzović, Elisabeth Damisch, Natalia Kulminskaya, Lukas Petrowitsch, Monika Oberer, Airi Palva, Nermina Malanović, Jeroen Codée, Walter Keller, Isabel Usón, Tea Pavkov-Keller

**Affiliations:** ^a^Institute of Molecular Biosciences, University of Graz, Graz, Austria 8010; ^b^Structural Biology Unit, Institute of Molecular Biology of Barcelona, Spanish National Research Council, Barcelona 08028, Spain; ^c^Laboratory of Computer-Aided Molecular Design, Division of Medicinal Chemistry, Otto-Loewi Research Center, Medical University of Graz, Graz, Austria 8010; ^d^Department of Bio-Organic Synthesis, Leiden Institute of Chemistry, Leiden University, Leiden 2333, The Netherlands; ^e^Department of Basic Veterinary Sciences, Division of Microbiology and Epidemiology, University of Helsinki, Helsinki 00100, Finland; ^f^Field of Excellence BioHealth, University of Graz, Graz 8010, Austria; ^g^BioTechMed-Graz, University of Graz, Graz 8010, Austria; ^h^Institució Catalana de Recerca i Estudis Avançats, Barcelona 08003, Spain

**Keywords:** S-layers, self-assembly, Lactobacilli, lipoteichoic acid, protein structure

## Abstract

S-layers are crystalline arrays found on bacterial and archaeal cells. *Lactobacillus* is a diverse family of bacteria known especially for potential gut health benefits. This study focuses on the S-layer proteins from *Lactobacillus acidophilus* and *Lactobacillus amylovorus* common in the mammalian gut. Atomic resolution structures of *Lactobacillus* S-layer proteins SlpA and SlpX exhibit domain swapping, and the obtained assembly model of the main S-layer protein SlpA aligns well with prior electron microscopy and mutagenesis data. The S-layer’s pore size suggests a protective role, with charged areas aiding adhesion. A highly similar domain organization and interaction network are observed across the *Lactobacillus* genus. Interaction studies revealed conserved binding areas specific for attachment to teichoic acids. The structure of the SlpA S-layer and the suggested incorporation of SlpX as well as its interaction with teichoic acids lay the foundation for deciphering its role in immune responses and for developing effective treatments for a variety of infectious and bacteria-mediated inflammation processes, opening opportunities for targeted engineering of the S-layer or lactobacilli bacteria in general.

## Significance

S-layer proteins (SLPs) are self-assembling, crystalline proteins coating the cell surfaces of many prokaryotes. This study presents experimental atomic resolution structures of lactobacilli SLPs, deriving functional insight into key probiotic *Lactobacillus* strains. The structures of SlpA and SlpX proteins highlight the domain swapping critical for SlpX integration, particularly in response to environmental stress. Two binding regions are identified as crucial for attachment of the S-layer to (lipo)teichoic acid. The structure of assembled S-layer provides a foundation for employing (designed) SLPs as a therapeutic agent in the treatment of inflammatory diseases. Additionally, it opens broad avenues for the use of SLPs in vaccine development and in crafting nanostructures with tailored properties, including those designed for targeted drug delivery.

Surface-layer proteins (S-layer proteins, SLPs) represent one of the most abundant cellular proteins found in prokaryotes (1). They self-assemble in a two-dimensional paracrystalline symmetrical manner, forming the outermost layer of the cell envelope (2, 3). Strong promoters control the metabolically highly expensive production of SLPs, thus highlighting their importance for bacterial survival (4). Considering their exposure on the surface, S-layers serve as first contact to the environment, playing distinctive roles in adhesion properties of cells, biofilm formation, and interactions with, e.g., the human immune system (5). Due to the ability of SLPs to self-assemble in a regular array, great application potential has emerged in biotechnology, nanotechnology, and medicine (3, 6, 7).

Various bacteria of the human microbiota, including the probiotic *Lactobacillus acidophilus,* contain S-layers and are considered harmless, safe, and beneficial for humans. Different strains of probiotic lactobacilli have been associated with other health benefits, ranging from supporting immune function (8) to promoting a healthy balance of intestinal flora when consumed (9). These bacteria may not only be naturally present in our digestive, urinary, and genital systems but also find their way into everyday foods such as yogurt (10). In particular, some strains of *Lactobacillus acidophilus* have been associated with assisting digestion, boosting the immune system, and helping to treat or prevent various conditions, including diarrhea, vaginal infections, and irritable bowel syndrome (11).

The S-layer of *L. acidophilus* and its closely related strain, *L. amylovorus*, are essential for its beneficial probiotic effect, as it allows colonization of the intestine and is responsible for its immunomodulatory properties (12–15). To date, limited experimental structural information on assembled S-layers is available (*SI Appendix*, Table S1), with no high-resolution structural information regarding lactobacilli SLPs. The SlpA protein (~46 kDa) is the main structural component of *Lactobacillus acidophilus* ATCC 4356 S-layer (16), built of two domains. The N-terminal region is involved in the self-assembly of the S-layer, whereas the C-terminal part is involved in the attachment to the cell wall (7, 17, 18). Two other S-layer proteins identified in this strain are SlpB (~47 kDa), whose expression is silenced, and SlpX (~51 kDa), which is probably involved in cell integrity (19). For *L. acidophilus* NCFM, SlpX is reported to built 10% of the S-layer under physiological conditions (20) and increases up to 40% under environmental stress (21). SlpA also represents a promising therapeutic agent for intestinal diseases since it induces immune responses, influences genes associated with colorectal cancer, and maintains the gut microbiome (22).

The *Lactobacillus* S-layers are anchored to the cell wall through interaction with teichoic acids (TA), either lipoteichoic acid (LTA) or wall teichoic acid (WTA) (17). Gram-positive TA are made up of repeating poly-glycerol phosphate (GroP) (23) or poly-ribitol phosphate subunits, often containing glycosyl or D-alanyl (D-Ala) esters (24). The chain length, the degree of substitution, and the quantity of LTA or WTA differ significantly between *Lactobacillus* strains and Gram-positives in general (24). Being a distinctive bacterial signature of gram-positive bacteria, including pathogenic *Staphylococcus aureus,* which is the leading cause of hospital-acquired infections (25), LTA also contributes significantly to the pathogenicity of bacteria (26–29). LTA is released from bacterial cells during growth but predominantly after antibiotic treatment due to bacteriolysis induced by lysozyme or beta-lactam antibiotics. As LTA binds nonspecifically to targets such as membrane phospholipids or, specifically, to Cluster of Differentiation-14 (CD14) and Toll-like receptors, it causes a signaling cascade in inflammation, e.g., production of proinflammatory cytokines such as tumor necrosis factor (TNF)-α (30).

Since lactobacilli have widespread prophylactic and therapeutic applications in numerous diseases (skin diseases, intestinal diseases such as inflammatory bowel disease, IBD, cancer, and adiposity) (29, 31), there is an urgent demand to enhance their therapeutic application. For this, structural information on *Lactobacillus* SLPs is essential. Here, we present atomic information on *Lactobacillus* S-layer proteins, SlpA and SlpX, including their assembly to a functional S-layer. We describe protein–protein interfaces, surface exposed areas, and pores formed upon S-layer formation and characterize their binding to TA. Furthermore, we show that the same domain organization and interaction network can be observed across the *Lactobacillus* genus.

## Results

### *Lactobacillus* SLP Contains Two Functional Regions.

**Fig. 1.**
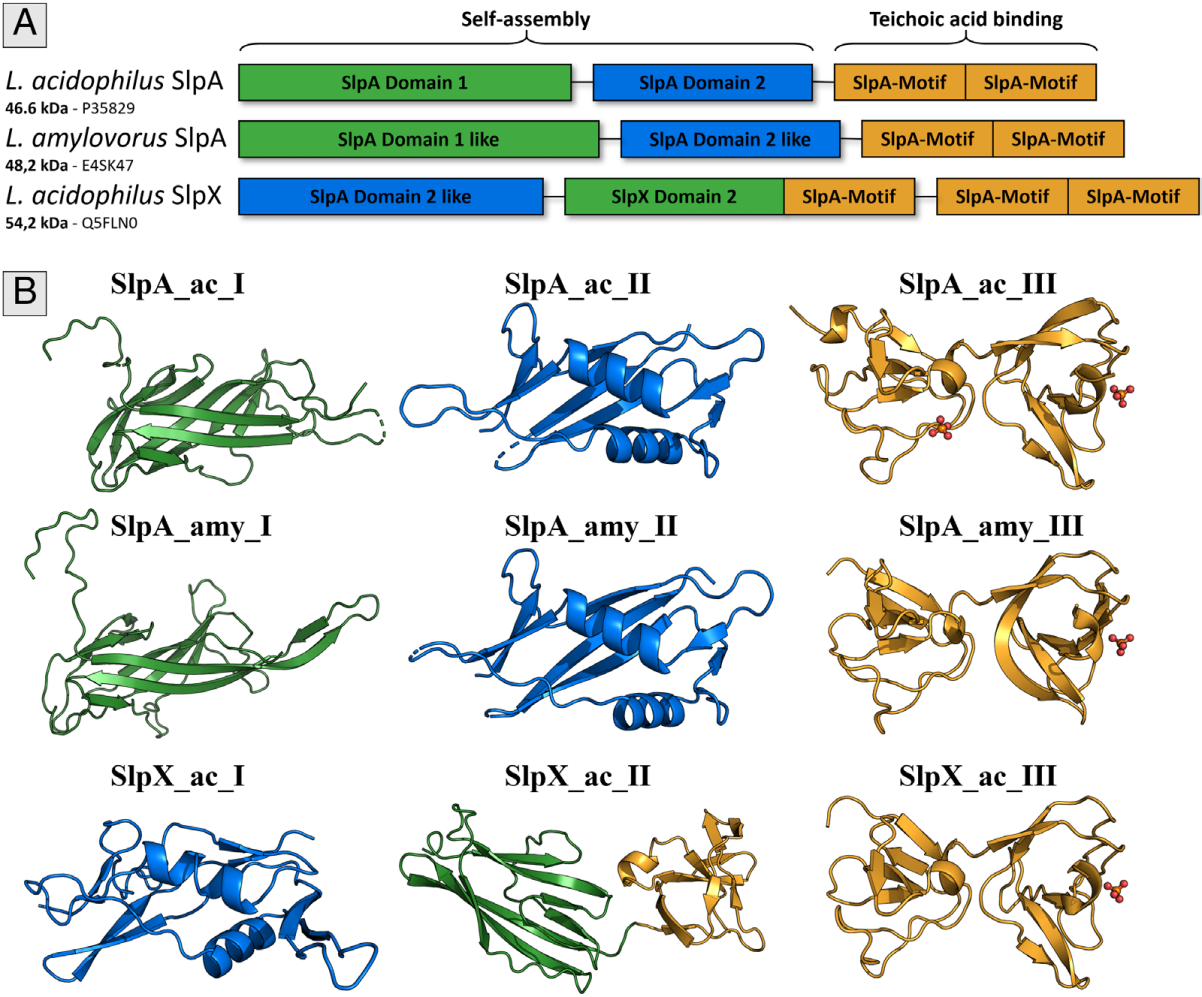
Domain arrangement and crystal structures of the S-layer proteins SlpA and SlpX of *L. acidophilus* and SlpA of *L. amylovorus.* (*A*) Schematic representation of the functional domain structure. Color-coded according to similar structural features. (*B*) Ribbon representation of individual domains as observed for S-layer proteins SlpA_ac, SlpA_amy, and SlpX_ac. Domains are color-coded according to the fold of the functional domains as in *A*. Phosphate ions are shown as spheres.

This study focuses on the proteins SlpA and SlpX from *Lactobacillus acidophilus* (SlpA_ac, SlpX_ac) and SlpA from *Lactobacillus amylovorus* (SlpA_amy). We determined the atomic resolution structures of all functional domains (I-III from N- to C-termini) present in the three S-layer proteins SlpA_ac, SlpX_ac, and SlpA_amy (Fig. 1 and *SI Appendix*, Tables S1 and S2). The arrangement and architecture of SlpA_ac and SlpA_amy are highly similar. The N-terminal part, exhibiting the first two domains (SlpA_I and SlpA_II), is known to be responsible for the self-assembly of the S-layer protein into a regular lattice (17). In SlpA_ac_I, the first ~20 N-terminal amino acids are not structured and reach out from the protein. According to a Foldseek Search (32) against the AlphaFold database (AFDB50), both domains show the best score with domain folds found in proteins from *Lactobacillus.* The search of the first domain SlpA_ac_I (7QLE) shows all top hits exclusively from *Lactobacillus* with E-values ranging from 3.2e^−17^ to 5.25e^−6^. The first hit which is not related to *Lactobacillus* has an E-value of 3.19e^−1^. This is also true for the second domain SlpA_ac_II (7QFL) with *Lactobacillus* top hits exhibiting E-values ranging from 5.89e^−16^ to 2.21e^−7^, whereas the first non-*Lactobacillus* hit has an E-value of 7.46e^−2^. The amino acid sequence of the C-terminal SlpA_III domain is sequentially conserved among *Lactobacillus*. It consists of two adjacent repeats of the “SlpA-motif,” the “Surface layer protein A motif/domain” (InterPro: IPR024968) (33). This domain is involved in the binding to TAs (teichoic acid binding domain; TAB domain), one of the components of the bacterial cell wall (7, 17, 18).

The full-length SlpX_ac shows differences in structure and domain arrangement compared to the SlpA architecture. SlpX_ac_I accommodates a similar fold to both SlpA_II, with SlpX_ac_I being slightly larger. The middle domain of SlpX, SlpX_ac_II, exhibits a SlpA_I-like domain fold and an additional repeat of the SlpA motif. SlpX_ac_III is structurally highly conserved compared to SlpA_ac_III and SlpA_amy_III, whereas the sequence is less conserved (37 to 41% identity, 52 to 56% similarity).

### Self-assembly of the SlpA_ac S-layer.

**Fig. 2.**
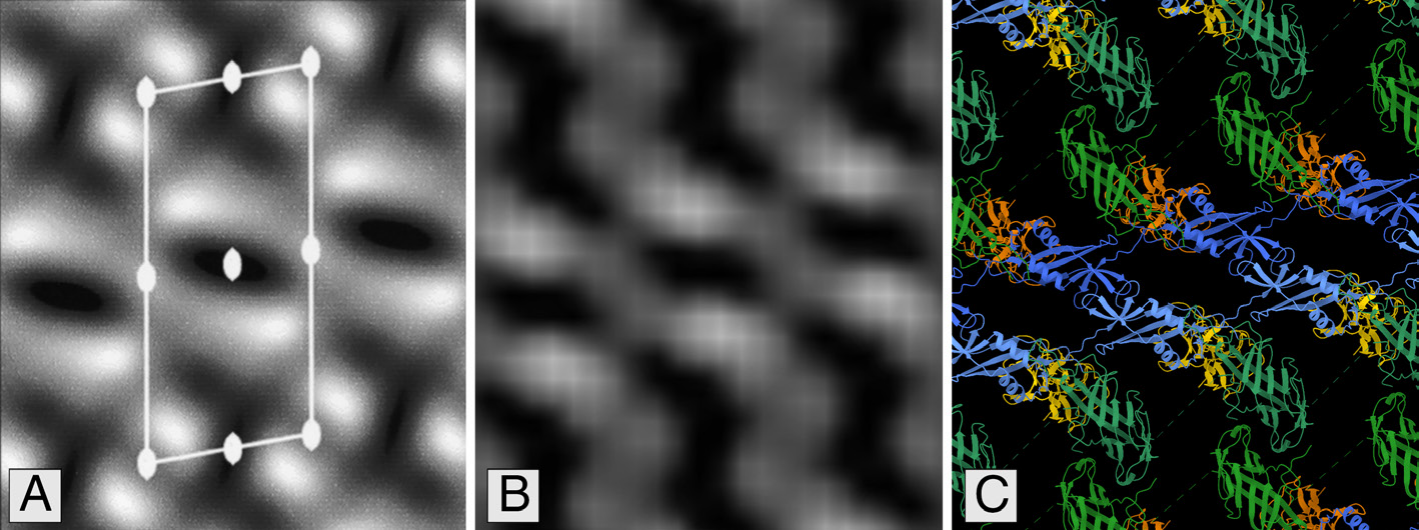
Comparison between available EM data and the proposed S-layer model. (*A*) Experimental projection map of the SlpA assembly layer as reported by Smit et al. (34) (*B*) calculated density map of the proposed assembled S-layer with pdb2mrc in ChimeraX (35) and (*C*) ribbon representation of the proposed S-layer model. SlpA_ac_I is shown in green, SlpA_ac_II in blue, and SlpA_ac_III in orange. One full-length chain is shown in slightly brighter colors.

The native S-layer protein from *L. acidophilus* ATCC4356 cells shows a *p*2 symmetry with lattice parameters a = 118 Å, b = 53 Å, γ = 102° (34). Since the S-layer in *L. acidophilus* predominantly consists of the SlpA protein, the proposed self-assembly model focuses on the interactions between the SlpA units. Combining our single-domain high-resolution crystal structures, evidence on the occurring crystal contacts, ab initio complex structure prediction models (*SI Appendix*, Fig. S1), existing mutagenesis data (*SI Appendix*, Fig. S2), and the available electron microscopy projection maps (34) we propose a detailed assembly model for the SlpA S-layer of *L. acidophilus* (Fig. 2 and *SI Appendix*, Fig. S3).

**Fig. 3.**
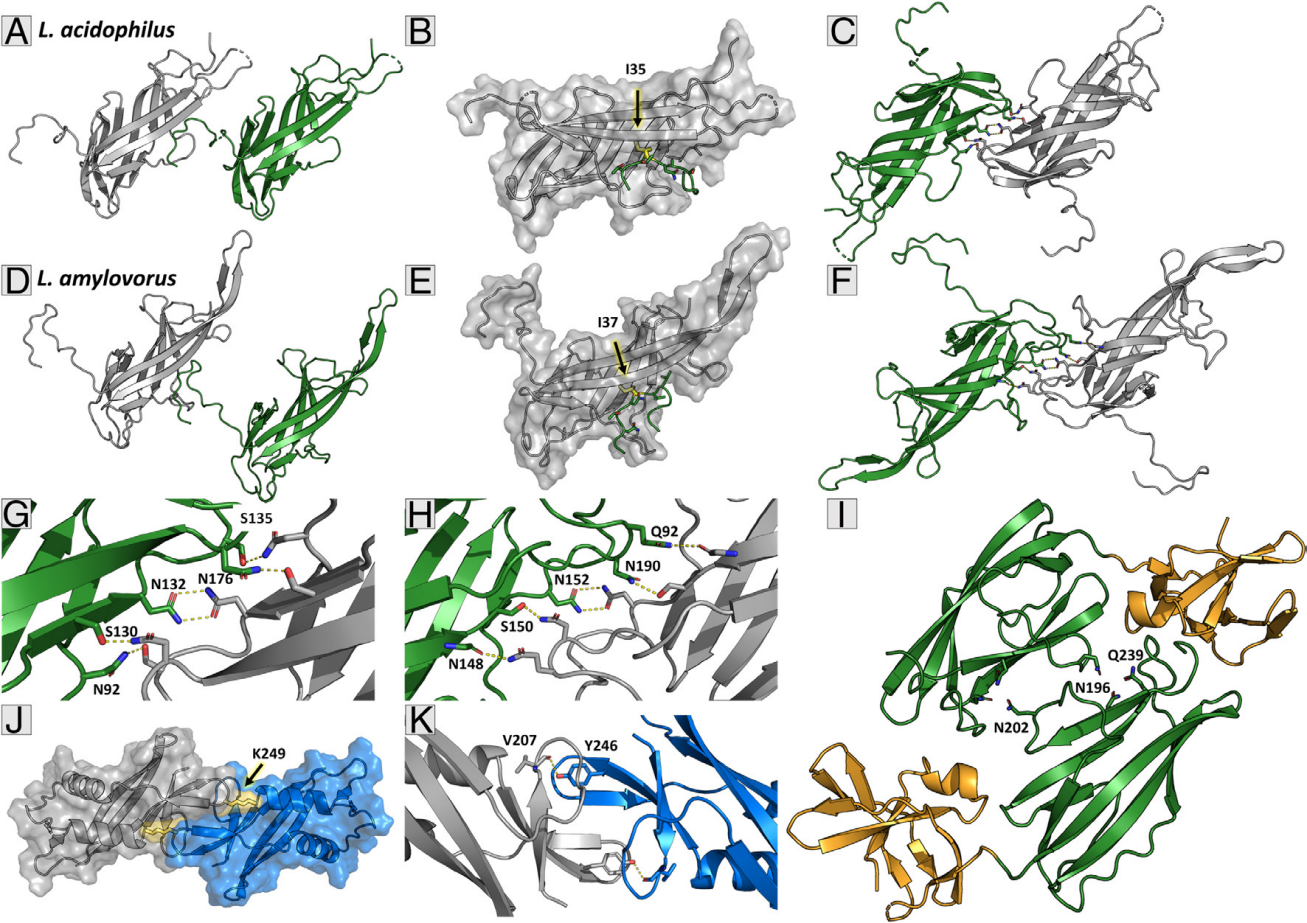
Experimentally observed interactions within SlpA_ac, SlpA_amy, and SlpX_ac. (*A*) The N-terminal tail of SlpA_ac_I (dark green) protrudes into a hydrophobic cleft of the neighboring SlpA_ac_I molecule (gray). (*B*) The neighboring SlpA_ac_I molecule is shown in surface representation, with the N-terminal tail where the conserved isoleucine (yellow) is situated. (*C*) The symmetrical *p*2 interaction between two SlpA_ac_I domains, responsible for the expansion of the S-layer in one direction. (*D*–*F*) The same mode of action and interfaces are found for SlpA_amy_I as in *A*–*C* for SlpA_ac_I. (*G* and *H*) The residues responsible for the interaction are highly conserved among lactobacilli (N92, S130, N132, N176, S135 in SlpA_ac_I and Q92, N148, S150, N152, and N190 in SlpA_amy_I). (*I*) The crystal structure of SlpX_ac_II (7QFJ) shows a dimer interface, corresponding to the interface observed in SlpA_ac_I (7QLE), indicating a possible homodimer of SlpX within the native S-layer. Residues N96, N202, and Q239 in SlpX_ac_II are located at the interface and form hydrogen bonds. The additional 3rd SlpA-motif (orange) is a part of the SlpX_ac_II, yet is not directly involved in this interaction. (*J*) Interaction between two SlpA_II_ac domains present in the crystal structure (PDB: 8BT9) leads to a dimer formation and a *p2* symmetry of the SlpA-assembly. Lys249 (yellow) reaches out for the next molecule and forms hydrophobic interactions. (*K*) Zoom of the interface of two SlpA_II_ac domains, stabilized by the interactions between Tyr246 and the backbone of Val207.

**Fig. 4.**
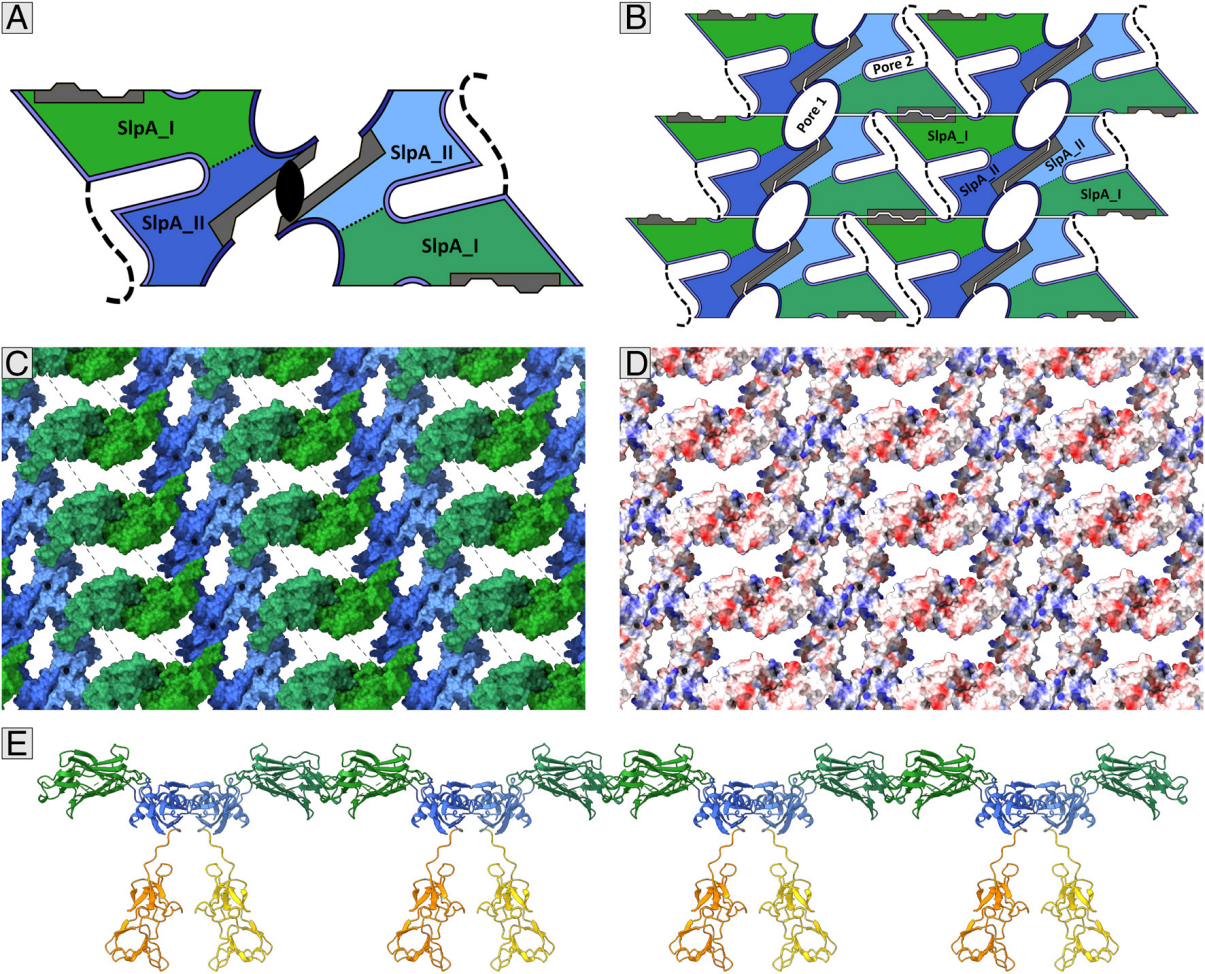
S-layer self-assembly model of SlpA_ac. (*A*) SlpA homodimer containing self-assembly domains I and II, shown as a tile, is a fundamental building block of the 2D assembly. This dimer is formed by the interaction of two SlpA_II domains (blue), showing a twofold *p*2 symmetry. The interfaces are colored gray. SlpA_III is not involved in the self-assembly, faces toward the cell surface, and is not depicted. (*B*) The 2D SlpA layer is assembled by repetition of tiles, consisting of one SlpA dimer per tile. Tiles are connected by the interaction between two SlpA_I domains (green) in one direction and by the N terminus to a neighboring SlpA_I domain in the perpendicular direction (dashed line). (*C*) The domains SlpA_I (green) and SlpA_II (blue) form the 2D assembly. Domains are shown as surface representation, with the flexible N terminus as a dashed line. The view of the S-layer is from the top. This part is exposed to the environment. (*D*) Surface representation of the outer surface of the S-layer, depicting the electrostatic potential with a color scale that varies from blue to red, representing the positive and negative potential, respectively. The orientation of the S-layer is the same in all panels. (*E*) Sideview of the assembled S-layer, including the SlpA_ac_III in orange/yellow (facing the cell wall).

The crystal structures of SlpA from *L. acidophilus* and *L. amylovorus* and observed crystal contacts reveal the self-assembly formation. In the SlpA_ac_I crystal structure (PDB: 7QLD, Fig. 3 *A* and *B*), the interaction between two SlpA_ac_I is present within the crystal packing, and the N-terminal tail extends far away from the main fold and reaches into a hydrophobic cleft of the neighboring molecule. A conserved isoleucine at the N terminus sits within this cleft. This appears to be a preserved mechanism, as the structural predictions of closely related strains demonstrate (*SI Appendix*, Fig. S4). A second interaction was observed within this crystal structure, a symmetrical homodimer interaction spanning from Asn92 to Asn176 (Fig. 3 *C* and *G*). Both interactions were also present in the experimental crystal structure of SlpA_amy_I (PDB: 8Q1O, Fig. 3 *D*–*F* and *H*), and the symmetrical *p2* interface was also present to some extent in the SlpA_I-like domain fold exhibited by SlpX_ac_II (Fig. 3*I*). This indicates that the lattice expands in one direction by interactions between two SlpA_I domains and in the other direction by positioning the N-terminal tail of SlpA_I within the cleft of a neighboring SlpA_I (Figs. 3*A* and 4*A*). In addition, our crystal structure of SlpA_ac_II (PDB: 8BT9) suggests on principle the SLP dimerization along SlpA_II involving Tyr246 and a loop region that spans from Asp206 to Gly210 (Fig. 3 *J* and *K*). This was also observed by AlphaFold multimer predictions (*SI Appendix*, Fig. S1).

Combining all these mentioned interactions and taking into account the flexibility of the linker regions, we propose the organization of the fully assembled SlpA_ac S-layer with the *p*2 lattice symmetry. One basic tile is composed of the homodimer of the full-length SlpA, dimerized at the SlpA_II domain (Fig. 4*A* and *SI Appendix*, Fig. S5). The assembly is propagated in one direction by alternating SlpA_I dimers and SlpA_II dimers and in the other by the pore spanning N terminus of SlpA_I (Fig. 4*B* and *SI Appendix*, Fig. S5). In this model, each SlpA unit contributes to two functionally separated parts of the assembly layer. The self-assembly region (SA) is formed by domains SlpA_I and SlpA_II, building a 2D crystalline network that forms the exposed surface of the SlpA layer (Fig. 4*E*). SlpA_III, the teichoic acid binding domain (TAB), is not involved in the self-assembly and protrudes inward to the bacterial cell (Fig. 4*E*).

In this organization, the exposed surface exhibits regions with mainly positive and negative charges (Fig. 4*D*). Furthermore, the SlpA_ac S-layer forms two pores: a smaller pore 1 with an elliptical shape and dimensions ~16 Å × ~26 Å and a larger pore 2 with an X-shape and dimensions ~14 Å × ~60 Å (Fig. 4 and *SI Appendix*, Fig. S6). The vertical pore height ranges between ~20 Å and ~30 Å. Both pores account for 20% of the total S-layer surface facing the environment. Regarding a possible function as a molecular sieve, the narrow character of both pores indicates a limitation in size of 14 Å in diameter for molecules passing through the S-layer. The surface of pore 1 is mostly uncharged, in contrast to pore 2 showing negatively and positively charged areas. Furthermore, the flexible part of the N terminus (aa 40 to 49) traverses pore 2 (Fig. 4*C*), very likely further reducing the dimensions of the pore.

### The TAB Domain Contains Two Putative TA Binding Regions.

**Fig. 5.**
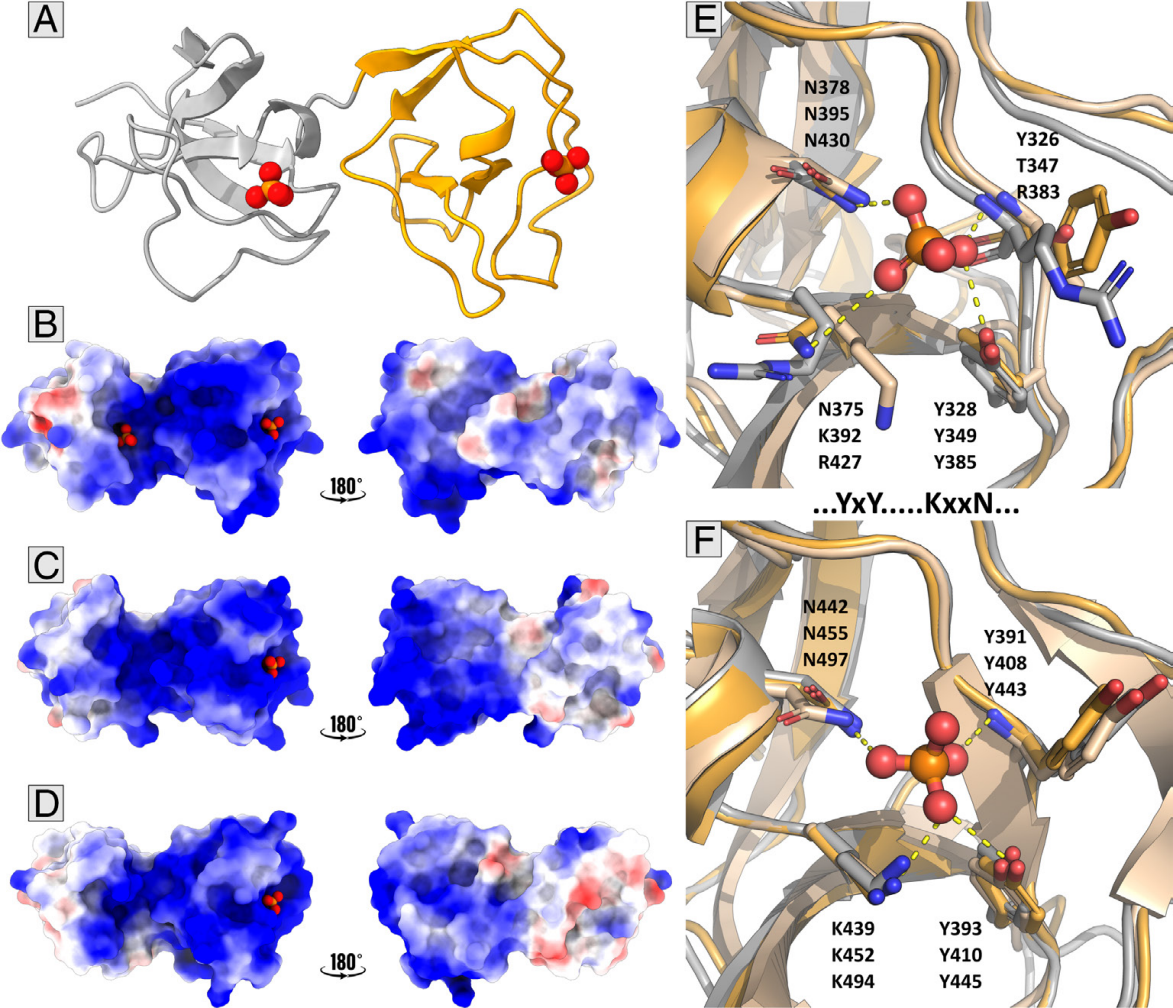
Conserved TAB domain with phosphate-binding sites. (*A*) The TAB domain overall structure consists of two SlpA motifs, gray and orange. Phosphates are shown as balls. (*B*–*D*) Surface representation of the TAB domains (*B*: SlpA_ac_III, *C*: SlpA_amy_III, *D*: SlpX_ac_III), depicting the electrostatic potential (blue to red, representing the positive and negative potential). (*E*) Superposition of the N-terminal phosphate-binding site of the three crystal structures and (*F*) of the C-terminal site: SlpA_ac_III (orange), SlpA_amy_III (ochre), and SlpX_ac_III (gray). In *E*, only in SlpA_ac_III, a phosphate is bound in the crystal structure. In *F*, a phosphate is bound to all three structures at the same position but is only shown for SlpA_ac_III (analog to *A*). Residues involved in the binding are shown as sticks and are labeled from top to bottom according to SlpA_ac, SlpA_amy, and SlpX_ac.

Following on the previously shown interaction of LTA with the C-terminal region of SlpA_ac corresponding to the TAB domain (17, 18), we performed sequential and structural analysis (Figs. 1 and 5 and *SI Appendix*, Fig. S8) with TAB domains of SlpA_ac, SlpA_amy, and SlpX_ac, in combination with experimental interaction studies using LTA and TA fragments. The crystal structures of the TAB domains (SlpA_ac_III, SlpA_amy_III, and SlpX_ac_III) reveal high structural similarities (Fig. 5 and *SI Appendix*, Fig. S7). The general structure of the TAB domain consists of two cell wall binding motifs (SlpA_motif) which are connected by a short linker and arranged as an internal homodimer (Fig. 5*A*). Mapping of the surface charges indicates a large, highly positively charged region within the interface of the two motifs that extends to the C-terminal part of the protein (Fig. 5 *B*–*D*). Two phosphate-binding sites are observed in the experimental structure of SlpA_ac_III (8ALU), situated within the positively charged cleft and at the corresponding position in the second SlpA motif (Fig. 5 *A* and *B*). The four residues observed in the vicinity of phosphates are conserved (Fig. 5 *E* and *F* and *SI Appendix*, Fig. S8) with the proposed binding motif of YxY….KxxN. These residues are not conserved in the third SlpA-motif, which is present in SlpX_ac_II.

### Attachment of the S-layer to the Cell Wall.

**Fig. 6.**
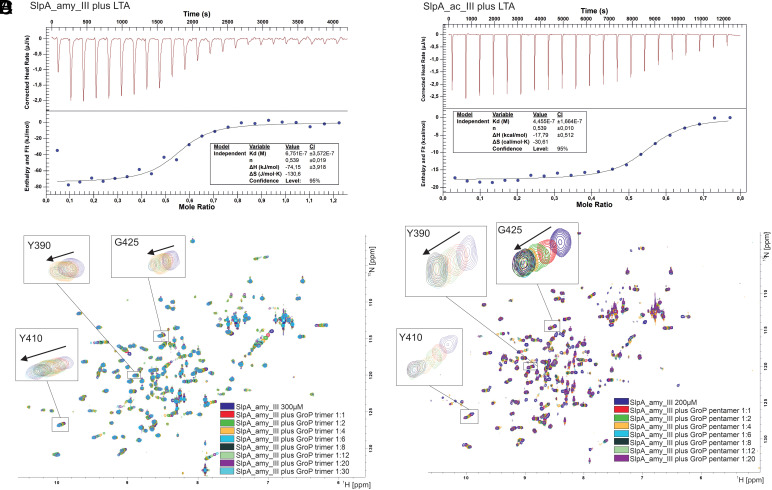
Characterization of the binding of SlpA TAB domain to the TA. (*A*) Binding of LTA to SlpA_amy_III and (*B*) SlpA_ac_III as observed by ITC measurements. CSP NMR experiments of SlpA_amy_III upon addition of (*C*) GroP trimer (ratios 1:1-1:30), and (*D*) GroP pentamer (ratios 1:1-1:20).

For elucidating SlpA interactions with the cell wall, we performed binding studies with the TAB domains and commercially available heterogenous LTA from *Bacillus subtilis*, as well as synthesized TA fragments consisting of glycerol-3-phosphate (GroP) subunits (36, 37). The experimental characterization of the LTA binding to the SlpA anchoring domain of gram-positive lactic acid bacterium *Lentilactobacillus buchneri* CD034 showed a 45 pN binding force typically associated with ligand–receptor interactions (23). We observed binding of the heterogenous *B. subtilis* LTA, containing on average 30 repeating glycerol phosphate units, with dissociation constants in the µM range via isothermal titration calorimetry (ITC). With NMR titration experiments, dissociation constants of 3.5 mM and 0.5 mM are determined for GroP trimer and GroP pentamer, respectively (Fig. 6 and *SI Appendix*, Tables S3 and S4), indicating that longer GroP fragments increase binding strength. The binding ratio, however, indicates that more protein can bind to the LTA, forming bigger complexes. In nature, LTA is embedded in the peptidoglycan, and fewer repeats are available for the interaction with the S-layer.

**Fig. 7.**
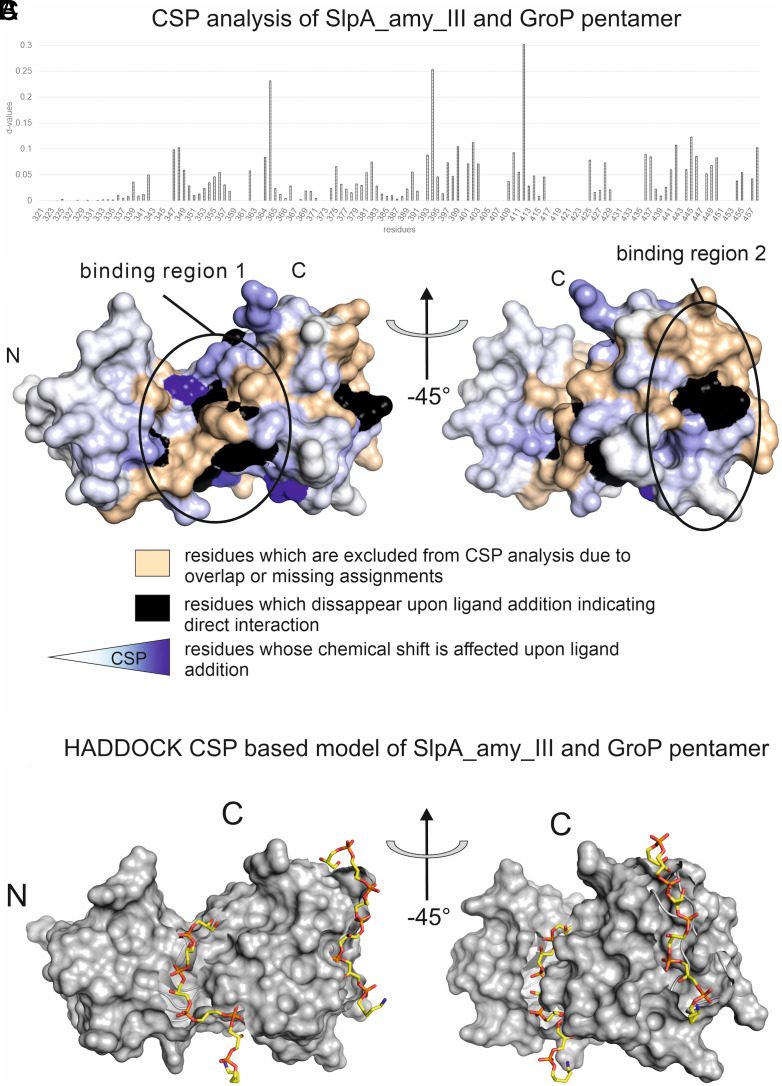
SlpA_amy_III forms two binding sites for GroP pentamer. (*A*) Graph representing the calculated d-values for residues of SlpA_amy_III upon addition of GroP pentamer. Higher d-values indicate location at or close to the binding site or indirect conformational changes upon ligand interaction. (*B*) Mapping of binding sites of GroP pentamer on the SlpA_amy_III structure. Residues are colored according to determined CSP derived d-values. Residues that disappear upon GroP pentamer addition are likely directly interacting with the ligand and are highlighted in black. Residues that experience chemical shift changes upon ligand interaction are colored according to a gradient: Blue represents strongly affected regions, which are highly influenced upon ligand interaction, and residues in white are unaffected. Residues shown in wheat are excluded from the analysis due to signal overlap or missing assignments. (*C*) Force-field minimized molecular model of SlpA_amy_III (gray surface) with both GroP pentamer ligands bound to TAB1 and TAB2 (C-atoms colored in yellow, P-atoms in orange). To generate the initial geometry, the experimentally derived CSPs were used as input for the calculation of protein–ligand models.

SlpA_amy_III NMR chemical shift analysis of GroP trimer and GroP pentamer titrations identified two areas corresponding to phosphate-binding sites: The binding area 1 (TAB1) forms an elongated cleft in the central region of the protein, and binding area 2 (TAB2) is located in the exposed region of the C-terminal part (Fig. 7 *A* and *B* and *SI Appendix*, Figs. S9 and S10). The main difference between the two binding regions is that both SlpA motifs contribute to the TAB1 located within the cleft of this domain. To obtain a molecular model of the SlpA_amy_III in complex with two GroP pentamers, we used a combination of molecular docking based on the NMR-derived chemical shift changes and classical molecular dynamics (MD) simulations (Fig. 7*C*). For both TAB1 and TAB2, reliable HADDOCK models (*SI Appendix*, Fig. S11) were obtained and minimized in energy.

Sequential analysis of TAB domains from different *Lactobacillus* strains (P38059, *L. helveticus*; A0A0PECX7L, *L. gallinarum*; Q09FM2 *L. crispatus*) reveals high conservation of residues located at the proposed TA-binding sites (*SI Appendix*, Fig. S8). There are non-SLP-proteins from different lactobacilli with a conserved TAB domain, like the surface protein “lysin” (Uniprot: A0A809KBB2, *L. acidophilus*), hyaluronidase (I7LAA9, *L. pasteurii*), N-acetylmuramidase (A0A4V6RD31, *L. intestinalis*), and a protein termed YSIRK-type signal peptide-containing (A0A6P1TWR6, *L. crispatus*). All mentioned proteins share the same predicted conserved fold and essential residues for the putative binding of TA binding in the TAB2. In contrast, in TAB1, the conserved asparagine is missing in non-surface-layer proteins. Taken together, we propose a general binding mode for lactobacilli SLP to the TA as well as for other non-surface-layer-related proteins exhibiting the conserved TAB domain.

## Discussion

S-layers are crystalline arrays commonly found on the surface of various bacterial species, including lactobacilli (*SI Appendix*, Fig. S13). Determining the structure of these abundant proteins in their natural lattice-like assembly on the bacterial surface is an important step in understanding their functions, properties, and interactions with the environment and the host. For lactobacilli, which are often used as probiotics, understanding the structure of the self-assembled S-layer can help manipulate their functionality to increase their effectiveness as probiotics (38).

**Fig. 8.**
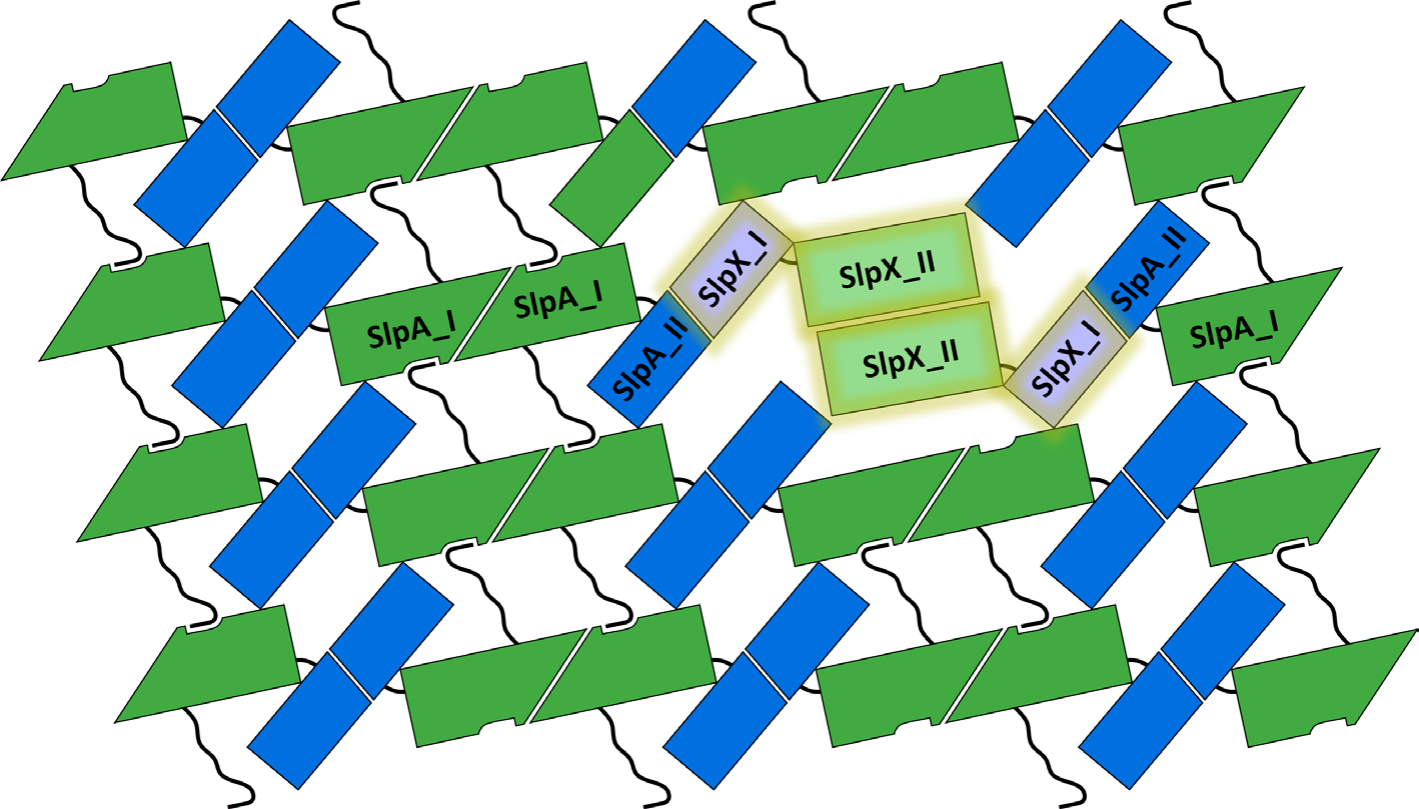
Model of proposed incorporation of SlpX into an existing SlpA S-layer. The putative SlpX homodimer is highlighted. Based on domain similarities between SlpX and SlpA, the following two interactions are proposed: SlpX_I (light blue) and SlpA_II (dark blue) and the homodimer of SlpX_II (light green). The cell-wall-binding domain of SlpX_III faces inward and is omitted in this figure for clarity.

The proposed assembled SlpA S-layer of *L. acidophilus* (Fig. 2 and *SI Appendix*, Figs. S3 and S13) was generated using a combination of high-resolution crystal structures of recombinant fragments including observed crystal contacts, as well as *ab initio* complex structure prediction models (*SI Appendix*, Fig. S1), available mutagenesis data (*SI Appendix*, Fig. S2), and published electron microscopy projection maps (34). The presented structural arrangement of the main protein SlpA in the self-assembled state shows *p2* symmetry and relatively small pores, suggesting a protective function of the S-layer, that serves as a physical barrier protecting the bacterial cell from external stressors, including harsh environmental conditions and host immune responses. SlpA represents up to 90% and SlpX up to 10% of the native S-layer that covers the bacterial cell (21). However, it is still unclear how SlpX is incorporated into the SlpA lattice, and how the different domain composition influences the interactions, pore size, and overall flexibility of the S-layer. In full-length SlpX, the first two domains are switched compared to SlpA, and our herein-presented crystal structure of SlpX_ac_II (7QFJ) shows a similar *p2* interface forming a homodimer like SlpA_ac_I with three strong hydrogen bonds (Fig. 3*I*). However, SlpX_ac_II contains an additional 3rd SlpA motif and an additional loop region, allowing for increased protein flexibility to incorporate into the assembled SlpA-SlpX S-layer. Likewise, SlpX_ac_I is structurally conserved (RMSD: 2.6 Å) to SlpA_ac_II, indicating the possible dimer formation as observed for SlpA_ac_I. Therefore, the SlpX homodimer might be incorporated within the assembled SlpA layer via the two described interfaces (Fig. 8). The TAB domain (SlpX_ac_III) would still face inward and anchor the S-layer to the cell wall analogously to the SlpA-only layer.

The proposed increased flexibility of SlpX might also be necessary for the self-assembly occurring at cell poles, where higher adaptability is required for effective cell division. Furthermore, different concentrations of bile salts influence the morphology and surface properties of the *L. acidophilus* S-layer (39). Integrating SlpX into the S-layer may affect pore dimensions, which is important during stress conditions like high osmotic pressure or salinity, where SlpX is known to be more prevalent (21, 39). SlpX is also associated with reduced growth rates, higher sensitivity to SDS, and greater bile resistance (19).

### Functional Domains of Lactobacilli SLPs.

**Fig. 9.**
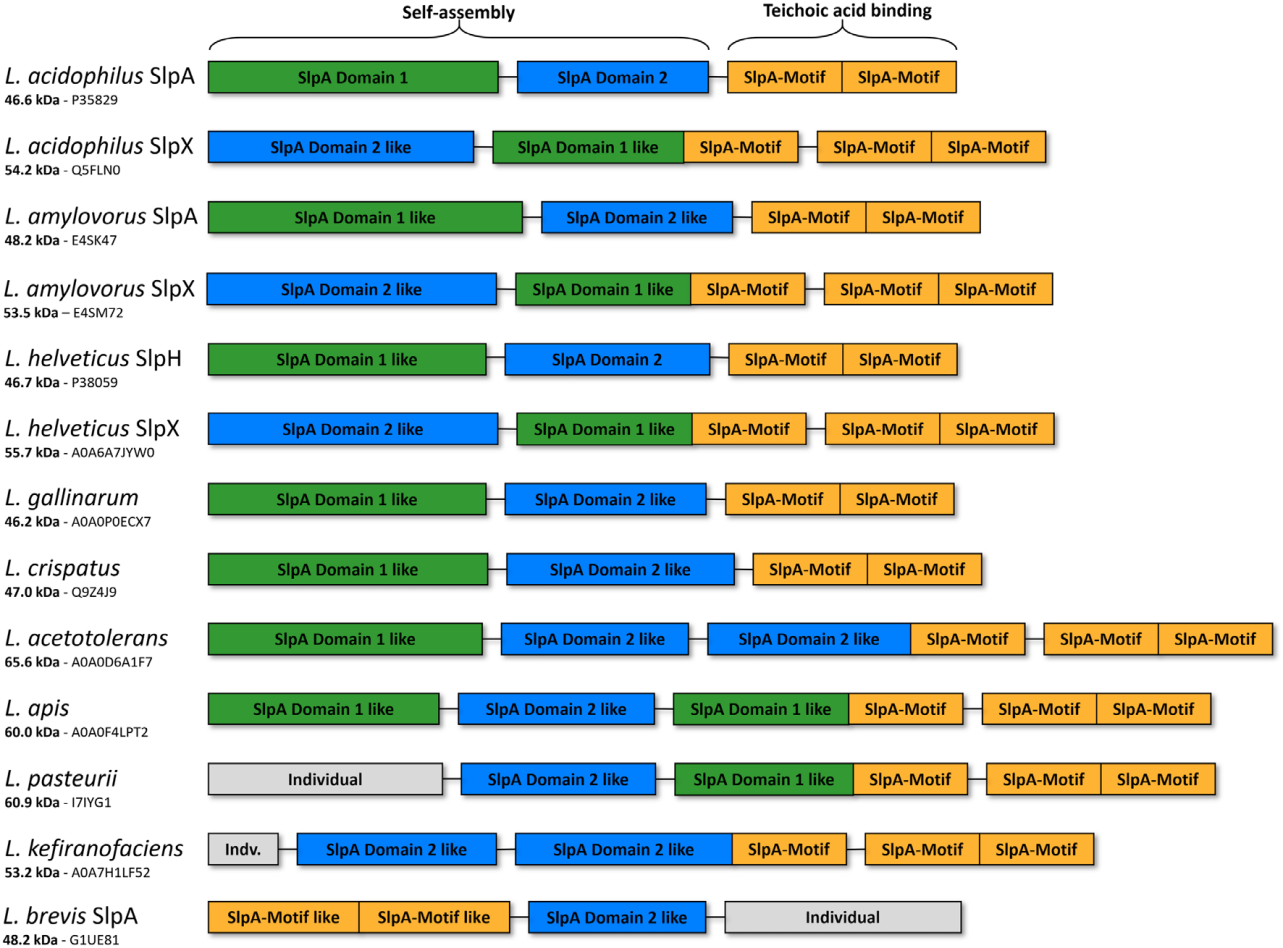
The domain organization of the S-layer protein family in different lactobacilli and levilactobacilli. The *Levilactobacillus brevis* SlpA protein is used as an example of the S-layer protein from the *Lactobacillaceae* family, where the TAB domain (orange) is at the N terminus.

The prediction of *slp* genes within the *Lactobacillaceae* family showed an interconnection between the two different modular S-layer protein domain organizations (40). The S-layers from *Lactobacillus* belong to a group where the TAB domain is placed at the C-terminus, whereas in *Levilactobacillus,* the TAB domain is at the N terminus. To evaluate whether the same self-assembly formation is possible for other S-layers from lactobacilli, we calculated Rosetta- and AlphaFold (41) models for several available SLP sequences. The sequence identity between individual lactobacilli S-layer proteins is low, except for the TAB domain. However, the Rosetta and AlphaFold models suggest the same fold and domain organization for most of the S-layers consisting of SlpA and SlpX proteins (Fig. 9). This indicates that even if the sequence similarity is low, the functional domains and likely the interactions within the self-assembled S-layers are highly conserved in this protein family. The predicted domain fold remains unchanged even in S-layer proteins with additional domains.

### TA Binding Areas Are Conserved among Lactobacilli.

Lactobacilli produce both LTA and WTA, although some strains contain only LTA, such as *L. rhamnosus* and *L. casei* (42). LTA is bound to the cell membrane via glycolipids, while WTA is covalently anchored to the peptidoglycan (PG) layer. WTA is, therefore, more likely to reach the cell surface beyond the peptidoglycan layer. In contrast, LTA must have a sufficient length to extend the PG layer, usually between 17 and 18 nm thick in lactobacilli and 20 to 80 nm thick in other gram-positive bacteria.

Considering the structure and composition of the *Lactobacillus* cell, we suggest that the S-layer can bind to both groups of teichoic acids, LTA and WTA, that have a chain structure with GroP as a repetitive unit, and show variations on substituents (23, 43, 44). It is not clear whether SlpA can also bind on WTA composed of ribitol phosphates as a repeating unit. The binding of TA to SlpA depends on the number of GroP subunits that reach beyond the PG layer. Our data show that longer TA fragments reveal stronger binding and substitutions like D-Ala or other bulky modifications might increase specificity. In each GroP repeat unit, there is a phosphate group that has a negative charge, which has the potential to attract positively charged components such as antimicrobial peptides (45). In this context, binding of the S-layer to LTA potentially shields bacteria from cationic molecules, reducing susceptibility to cationic antimicrobial peptides in certain lactobacilli (46).

### Immunological Importance of SlpA and LTA.

Many lactic acid bacteria are used as probiotics, which requires understanding whether and in what manner the S-layer and LTA impact their probiotic characteristics (5, 16). Given their location on the bacterial cell surface, S-layers are in direct contact with the host’s immune system. An interaction of the isolated SLP with Pattern Recognition Receptors (PRRs) such as C-lectin type receptors (CLTRs) has been reported. The isolated SlpA from *L. acidophilus* has been shown to interact with the human dendritic cell receptor DC-SIGN which plays a crucial role in the recognition and immune response (47, 48). However, the mode of binding is unclear (5, 16, 49) as well as at what level this binding influences the probiotic properties. Generally, recognition by DC-SIGN is driven by specific glycosylation patterns, and the glycosylation of SlpA from *L. acidophilus* is proposed (18, 47). The predicted N-glycosylation sites are situated mostly in surface-exposed areas of the proposed S-layer (*SI Appendix*, Fig. S12) making them accessible to the environment, whereas three sites are situated near the interaction interfaces between SlpA_ac monomers. Alternatively, the positively and negatively charged regions identified at the surface of the assembled SlpA might serve as a microbe-associated molecular pattern recognized by the host immune system’s PRRs.

SlpA and LTA induce contrary effects in intestinal diseases due to their interaction with the immune system. Whereas LTA causes proinflammatory immune reactions, (50, 51) SlpA has been shown to suppress inflammation processes and restore beneficial gut microbiomes (52). For instance, in the context of murine colitis treatment, isolated *L. acidophilus* and genetically modified *L. acidophilus* strains that selectively presented SLP on their surface as well as treatment with purified SLP exhibited significant therapeutic benefits (29, 31, 42, 53). This renders the SLP as a promising candidate for a therapeutic agent in treating inflammatory diseases and suggests its potential as a component or adjuvant for vaccines and in the design of nanostructures with the defined properties, such as for targeted drug delivery (54). The surface of probiotic strains, in particular SlpA and LTA, plays a crucial role in the treatment of colitis due to their specific interactions with the immune system (50). A detailed understanding of the construction of S-layers thus opens the path for genetic modification thereby regulating subsequent inflammation or anti-inflammatory immune responses. By altering specific binding sites or surface epitopes, it is now feasible to engineer strains that exhibit enhanced adhesion to the gastrointestinal lining, improve survival during storage and transit through the digestive tract, or show increased immunomodulatory effects.

SLP isolated from various lactobacilli exhibited a stimulatory effect in inducing the expression of human β-defensins. These peptides contribute to the host’s immune system as a first defense mechanism against pathogens. This induction occurred through activating the TLR-2 and the c-Jun N-terminal kinase signaling pathway. Thus, SLP contributes to host protection against infections (55). This may be beneficial in fighting other pathogens, for example, gram-negative bacteria like *Salmonella* (56). Furthermore, to reduce the proinflammatory effects of LTA, the TAB domain offers an approach to mitigating inflammatory responses. With now-available atomic resolution information on LTA binding regions, the engineering of the TAB domain for the improved binding of LTA from various sources will become possible.

In summary, the atomic structures of *Lactobacillus* S-layer proteins, SlpA and SlpX, along with the observed differences in their domain organization, enabled us to generate a self-assembly model for SlpA, the main SLP. This model aligns with prior studies and suggests a domain-swapping mechanism that enables the integration of SlpX into the S-layer (Fig. 8). This structure not only aids in identifying key surface areas for host interactions but also paves the way for using S-layer proteins or engineered lactobacilli in therapeutic applications, such as treating inflammatory diseases and vaccine development (56). Additionally, understanding the S-layer’s attachment to teichoic acids represents a foundation for strategies in combating infectious and bacteria-mediated inflammation processes for pathogenic bacteria containing LTA in the cell wall.

## Materials and Methods

### Cloning.

Full-length surface-layer genes of SlpA_amy of *Lactobacillus amylovorus* strain GRL 1112 and SlpA_ac and SlpX_ac of *Lactobacillus acidophilus* strain ATCC4356 were used to produce three functional fragments of each gene.

*L. acidophilus* SlpA was produced by amplification of *L. acidophilus* chromosomal DNA. The chromosomal DNA was purified from a culture of *L. acidophilus* ATCC4356 cells by a standard phenol-chloroform extraction. SlpX_ac full-length gene was purchased at Thermo Fisher via custom DNA synthesis service (protein sequence Uniprot Q5FLN0; DNA sequence was codon optimized for *Escherichia coli* expression).

All constructs listed in *SI Appendix*, Table S2 were either cloned in the vector pJC40 (ampR), pET-28b (kanR), or pET-28a(+)-TEV (kanR). Constructs in the pJC40 vector were cloned with NdeI and BamHI as restriction sites and N-terminal 10× HisTag, respectively. Constructs in both pET-28 vectors were cloned with NcoI and XhoI as restriction sites and a C-terminal 6× HisTag.

For gene amplification, specific primers (250 nM, each) (*SI Appendix*, Table S3), template DNA (10 ng), and HotFire Pol DNA Polymerase from Solis Biodyne (Tartu, Estonia) were used. A total of 30 cycles were performed according to the manufacturers. Amplified DNA fragments were purified with a 1% agarose gel extracted with the GeneJET Gel Extraction Kit (Thermo Fisher) according to the manufacturer’s protocol. The purified gene inserts and the corresponding vector were digested with the respective restriction enzymes, NdeI, BamHI, NcoI, and XhoI (New England Biolabs, Ipswich, United States) at 37 °C for 3 h. Ligation was carried out with a 30 fmol vector, 150 fmol insert, and T4 DN- Ligase (NEB) at 16 °C overnight.

### Transformation.

NEB Turbo Competent *E. coli* (High Efficiency) was used for plasmid amplification. First, 50 ng of plasmid DNA, 20 µL 5× KCM buffer, and 60 µL competent *E. coli* were mixed and incubated on ice for 30 min. The mixture was kept for 10 min at room temperature. Then, 500 µL of LB medium was added, and after incubation at 37 °C for 45 min, cells were plated on agar plates containing the appropriate antibiotics. The plasmid was purified after overnight cultures of the selected clones (Invisorb Spin Plasmid Mini Two, Stractec Molecular, Berlin). The plasmids were sent to Microsynth AG (Balgach, Switzerland) for sequencing.

For the expression of proteins, NEB Lemo21 (DE3) Competent *E. coli* was used. First, 100 ng of plasmid was added to 200 µL of competent cells. The mixture was incubated on ice for 30 min; a heat shock was then performed at 42 °C for 30 s. Subsequently, the cells were kept on ice for a further 5 min. After this period, 500 µL of LB medium was added, cells were incubated at 37 °C for 45 min, and the cells were seeded in agar plates containing the appropriate antibiotics.

### Expression.

*E. coli* Lemo21 cells carrying the respective plasmid were grown at 37 °C until they reached an OD600 of 0.6 with compatible antibiotics, ampicillin in the case of the pJC40 vector and kanamycin for pET28b and pET-28a(+)-TEV. After induction with 0.5 mM IPTG, the culture was incubated at 25 °C for 16 h. Cultures were harvested at 4,500 rpm (JA-10 rotor, Avanti centrifuge J-26 XP, both Beckman Coulter). The cell pellets were frozen at −20 °C for further use or directly used for lysis and purification.

### Expression of Seleno-methionine (SeMet) Labeled SlpA_amy_III.

SlpA_amy _III was expressed in *E. coli* B834 cells to incorporate seleno-methionine into the protein following the EMBL protocol for seleno-methionine expression (57). An overnight culture (ONC) with minimal methionine-supplemented medium was carried out at 37 °C. Seleno-methionine and methionine stock solutions (Molecular Dimensions, Maumee) were added to a final concentration of µg 50 g/mL each. Two 500 mL flasks were inoculated with ONC in a 1:100 ratio and incubated at 37 °C until an OD600 between 0.6 and 1. The flask contents were centrifuged and resuspended in a minimal medium without methionine for another 2 h. Seleno-methionine was added, and 30 min later, the culture was induced using 0.5 mM IPTG and cultured for another 5 h before harvesting.

### Protein Purification.

The recombinant protein was purified by immobilized metal affinity chromatography (IMAC) using a nickel-NTA coated column followed by size exclusion chromatography. The cell pellet was resuspended in 25 mL of a 50 mM phosphate buffer pH 8, 300 mM NaCl, and 50 mM imidazole. This suspension was sonicated with a Bandelin Sonoplus sonicator at 50% and five cycles for 25 min with constant cooling. The resulting suspension was centrifuged for 30 min at 4 °C and 18,000 rpm in the JA-25.50 rotor in Avanti J-26 XP, both Beckman Coulter (Brea). The supernatant was filtered Rotilabo fiber glass syringe filter (25 mm membrane) and Rotilabo syringe filter, PVDF (pore size 0.45 µm) from Carl Roth GmbH and Co. before loading samples onto a ÄKTA purifier system from GE Healthcare. The HisTrap FF affinity column 5 mL from GE Healthcare was equilibrated with the previously mentioned lysis/wash buffer mentioned below. According to the standard GE Healthcare HisTrap purification protocol, proteins were eluted during the elution buffer in a linear gradient with elution buffer (50 mM phosphate buffer pH 8, 300 mM NaCl, and 400 mM imidazole). After SDS-PAGE inspection, fractions containing protein of interest were pooled and concentrated using Amicon Ultra centrifugal filters (Millipore, Merck KGaA). Up to 10 mL was injected into the ÄKTA Prime system from GE Healthcare with a self-packed SEC column XK26/100 filled with Superdex 200 pg resin. The column was equilibrated with 20 mM HEPES pH 7 or 8, depending on the protein, and 100 mM NaCl. The collected fractions were concentrated as above. Concentrated purified proteins were aliquoted, shock frozen, and stored at –20 °C.

### Crystallization.

Crystallization experiments were performed using vapor diffusion sitting drop and in Swissci UVXPO 3 Lens crystallization plates (High Wycombe). Pipetting was carried out with an Oryx 8 robot (Douglas Instruments) with 30 µL of condition solution in the reservoir and drops of 0.5 µL protein; the concentration ranges from 10 mg/mL to 20 mg/mL, mixed with 0.5 µL screening solution. The SlpA_ac_II S115C mutants were cocrystallized with 1.5 mM HgCl_2_. An additional 0.1 µL of a 10-5 to 10-7 diluted seeding stock was applied for seeding experiments. The plates were incubated at 20 °C. Optimization experiments were pipetted manually in Crystal clear duo strips from Douglas Instruments with 70 µL of commercial screening solution in the reservoir and drops of 1.4 µ protein and condition in a 1:1 ratio. The commercially available screens used and the conditions for successful crystallization are described in *SI Appendix*, Table S4.

### Data Collection and Processing.

Crystals were frozen in liquid nitrogen using 5 to 15% glycerol as cryoprotectant. Data collection of all crystals was performed at 100 K. Crystal screening and data collection were carried out on the synchrotron beamlines at Elettra Beamline 11.2C (Trieste, Italy), Petra III DESY Beamline P11 (Hamburg, Germany), ESRF Beamlines ID23-1, ID23-2, MASSIF-1, and ID30B (Grenoble, France), and SLS X06SA (Villigen, Switzerland). Datasets were processed and scaled using the XDS program package (58) or the ISPyB Automatic data processing pipelines. Data collection and processing statistics of the best dataset for each truncation form are shown in *SI Appendix*, Table S5.

### Structures Solution, Refinement, and Analysis.

Since no structures of homologs were available, alternative approaches were chosen to overcome the phase problem. The structures of SlpA_amy_III and SlpA_ac I were solved by Se and Hg-SAD, respectively. The initial heavy atom positions, initial phases, and model were calculated with ShelXC/D/E (59). The structures of SlpX_ac_I and SlpA_amy_II were solved ab initio with ARCIMBOLDO_LITE (60). The calculations were run on an HTCondor Grid of 160 nodes. Fragment search was set to two polyalanine alpha helices of 14 residues following the eLLG criterion (61) in Phaser (62). After location and rigid body refinement of sets of two fragments, the best probes scored by LLG and ZSCORE were sent to expansion through density modification and map interpretation with SHELXE (59). Parameterization used a set of 15 cycles of density modification and polyserine autotracing.

All other structures were solved by molecular replacement using Phaser (62) and the corresponding templates (*SI Appendix*, Table S5). The preliminary models were manually rebuilt using Coot (63) and refined using phenix.refine (64). The Coulombic electrostatic potential (ESP) was calculated for the SlpA model of the assembled layer using ChimeraX (35). Structural superposition was performed using SSM Superposition within Coot. All structural images were prepared using PyMOL (65) and ChimeraX (35).

### Model of SlpA_ac, Assembled in 2D Crystal Lattice.

The molecular model of the 2D crystal lattice formed by SlpA_ac was created by combining two sets of predictions with features extracted from preexisting EM projection maps (34) (*SI Appendix*, Fig. S2) and observed crystal contacts from single-domain crystal structures. All predictions were made on an AlphaFold Multimer installation with full databases (66, 67) in standard configuration for prokaryotes.

The first set of models was calculated for a full-length SlpA_ac dimer (see *SI Appendix*, Table S6 for input sequences). Four out of five models showed consistent contact at the region of SlpA_ac_II, also present in the crystal structure (PDB 8BT9). The second set of models was calculated for the single-domain SlpA_ac_I dimer. Three of five models show a contact also present in both experimental crystal structures of SlpA_ac_I. Only the matching contacts were used for further investigation.

The SlpA_ac and SlpA_ac_I dimer models were combined by superposition of SlpA_ac_I, using the matchmaker command of ChimeraX (35). This leads to the crystal lattice’s minimal building unit, which was translated to create a lattice arrangement that is qualitatively compatible with the pattern of the preexisting EM density map (34). The flexible interdomain linkers can be used to adjust the arrangement to match the unit cell parameters determined by Smit et al. (34) a = 118 Å, b = 53 Å, γ = 102°.

### Ab Initio Model of Pore-forming Complex.

A complete pore-forming subsection of the 2D crystal lattice was predicted by calculating an ab initio model of a complex containing two full-length SlpA and two SlpA_II domains. Three of five ab initio models show a complex that forms a pore that is qualitatively compatible with the model of the 2D crystal created by superposition.

### Isothermal Titration Calorimetry.

The ITC measurements were performed using a NanoITC calorimeter (TA Instruments). The sample cell (190 μL) was loaded with SlpA_III (20 mM HEPES pH 8.0 and 100 mM NaCl). LTA from *B. subtilis* (Sigma) solutions were prepared in the same buffer. ITC measurements were conducted at 25 °C. The mixing rate was 250 to 300 rpm, SlpA_III concentration was 0.045 to 0.1 mM, and the concentration of the LTA was 0.28 to 0.3 mM. The injection volume was 1.96 µL using a titration interval of 180 to 600 s. Blank titration of LTA into the buffer and buffer into protein were performed. NanoAnalyze software from TA Instruments was used to analyze the obtained data. All binding curves including the thermodynamic parameters were obtained using the independent fitting model.

### NMR Sample Preparation.

For NMR experiments, *E. coli* BL21 DE cells containing the SlpA_amy_D3 gene were grown in minimal M9 media, including ^15^N-labeled (NH_4_)_2_SO_4_; for NMR backbone assignment, M9 minimal media supplemented with ^15^N-labeled (NH_4_)_2_SO_4_ and ^13^C labeled (NH_4_)_2_SO_4_ and ^13^C labeled glucose was used. Cells were grown with shaking (180 rpm) at 37 °C. Induction was achieved by adding 1 mM IPTG when OD_600_ reached 0.6 to 0.8. After induction, the culture was incubated at 20 °C overnight.

After overnight expression, cell cultures were centrifuged and the pellet dissolved in 20 mL of loading buffer containing 20 mM Tris, 300 mM sodium chloride, and 10 mM imidazole at pH 8. A protease inhibitor was added to the loading buffer. Cells were disrupted by sonication, and the lysate was centrifuged. The supernatant was loaded on a gravity column containing 2 mL of Ni-NTA agarose beads. The column was washed with 15 column volumes (CV) of loading buffer followed by five CV of the loading buffers containing 1 M sodium chloride and five CV of the loading buffer containing 20 mM imidazole. His-tagged proteins were eluted with five CV elution buffers containing 330 mM imidazole. The final purification step included purification by FPLC using a HiLoad 26/600 Superdex 75 pg column in 20 mM Tris, 300 mM sodium chloride, pH 8. The sample was dialyzed overnight in NMR buffer containing 20 mM ADA (N-(2-acetamido)iminodiacetic acid) and 50 mM sodium chloride, pH 6.5.

### NMR Backbone Assignment.

All NMR spectra were recorded on a Bruker Avance III 700 MHz spectrometer equipped with a cryogenically cooled 5 mm TCI probe using gradients on the z-axis at 25 °C. NMR samples were prepared in 90% H_2_O/10% D_2_O and measured in a 5 mm tube. As an NMR sample buffer, we used 20 mM ADA, 50 mM sodium chloride, and pH 6.5. For the assignment of the backbone resonances, standard triple resonance experiments were used: HNCO, HN(CA)CO, HNCACB, HN(CO)CA, HNCA, HN(CA)CO, and ^15^N-HSQC. Backbone assignments were carried out with CcpNMR 2.4.1. (68). NMR chemical shift assignments are deposited at BMRB (accession number: 52156).

### NMR Titration Experiments.

The GroP trimer and pentamer were synthetized as previously described (69, 70). For titrations, ligands were dissolved in ddH_2_O and added to ^15^N-labeled SlpA_amy_D3 in NMR buffer. GroP trimer titrations were carried out with a 300 µM SlpA_amy_D3 sample. Increasing amounts of GroP trimer were added, and ^15^N-HSQC experiments were recorded after each step. The final protein-to-ligand ratios were 1:1, 1:2, 1:4, 1:6, 1:8, 1:12, 1:20, and 1:30. The GroP pentamer was added to a 200 µM SlpA_amy_D3 resulting in final ratios of protein to ligand of 1:1, 1:2, 1:4, 1:6, 1:8, 1:12, and 1:20. Spectra were processed with NMRPipe (71) and analyzed using CcpNMR 2.4.1. (68). To detect the interface of the interaction between SlpA_amy_II and GroP pentamer and trimer, respectively, we used the formula described below. The calculated d-values give information about the degree of change of the chemical shift after the ligand addition. Peaks which disappear after addition of ligands or peaks located in the crowded middle region of the spectra may also be influenced by the binding but cannot be included in the calculation.

$$d=\sqrt{\frac{1}{2}\left[\delta H^{2}+(\alpha *\delta N{)}^{2}\right]},$$

δH/δN, chemical shift changes [ppm]; α, scaling factor α = 0.14, except for glycins α = 0.2.

### NMR Relaxation Experiments.

NMR relaxation experiments of SlpA_amy_III were recorded in a buffer containing 20 mM ADA, 50 mM sodium chloride, and pH 6.5. 15N T1 values were measured using the hsqct1etf3gpsi3d.2 bruker pulse program, with delay times of: 0.4, 2.0, 0.2, 1.5, 0.5, 0.7, 1.0, 0.3, 3.0, 3.5, 0.1, 0.8, 0.05 s. The ^15^N T2 values were measured using hsqct2etf3gpsi3d with varying delays of 0.68, 0.22, 0.229, 0.034, 0.204, 0.17, 0.114, 0.085, 0.237, 0.136, 0.051, 0.170, 0.102 s. The {^1^H}-^15^N heteronuclear NOEs were measured using the pulse sequence hsqcnoef3gpsi. Spectra were processed using NMRPipe and analyzed via CcpNMR 2.4.1. (68). The rotational correlation time was calculated using the formula shown below.

$$\tau c\approx \frac{1}{4\pi \nu N}\sqrt{6\frac{T1}{T2}}-7,$$

τc, rotational correlation time (s); νN, ^15^N resonance frequency (Hz); T1, ^15^N T1 relaxation time (s); T2, ^15^N T2 relaxation time (s).

### Calculation of Dissociation Constants by NMR.

The dissociation constants were calculated using CcpNMR 2.4. (66). For GroP pentamer and GroP trimer NMR titrations, the dissociation constant (Kd) was fitted for each amino acid individually with equation below and a mean Kd value was calculated (*SI Appendix*, Tables S7 and S8).

$$\Delta \delta_{\text{obs}}=\frac{\Delta \delta_{\text{max}}\left\{\left([P{]}_{t}+[L{]}_{t}+K_{d}\right)-\sqrt{{\left({\left[P\right]}_{t}+{\left[L\right]}_{t}+K_{d}\right)}^{2}-4{\left[P\right]}_{t}{\left[L\right]}_{t}}\right\}}{2{\left[P\right]}_{t}},$$

$\Delta \delta_{\mathrm{obs}}$, change in observed shift; $\Delta \delta_{\max}$, maximum shift change on saturation; ${\left[P\right]}_{t}$, total protein concentration; ${\left[L\right]}_{t}$, total ligand concentration; $K_{d}$, dissociation constant.

### Calculation of the SlpA_amy_III-GroP Pentamer HADDOCK Models.

To obtain a molecular model of the complexes between the TAB domain (SlpA_amy_III), PDB 7QEH and the GroP multimers, we used a combination of molecular docking and classical MD simulations. The NMR-derived chemical shift changes were used as an input for calculating SlpA_amy_III-GroP pentamer complexes via the software HADDOCK 2.4 (72). A better convergence was reached when each TAB domain binding site was modeled separately. The CSP data derived from the NMR-titrations were used to drive the docking process, and the residues with the strongest chemical shift changes as well as disappearing residues were used to define the active protein interactions. Binding site 1 active residues were 346, 363, 364, 394, 400, 412, 430, 431, 432, 434, and 435. Binding site 1 passive residues were 341, 365, 366, 380, 396, 397, 398, 399, 402, 427, 429, 440, 445, 446, 438, 439, 415, 413, 383, 384, 385, 386, 387, 389, 357, and 358. Binding site 2 active residues were 407, 408, 411, 410, and 455. Binding site 2 passive residues were 427, 428, 439, and 415. For data interpretation and figure generation, we used the cluster with the lowest overall HADDOCK-score and the number 1 best structure (*SI Appendix*, Table S9).

### MD Simulations.

The initial coordinates for the SlpA_amy_III/GroP pentamers complex were obtained with HADDOCK. The Cartesian coordinates of SlpA_amy_III were obtained from the X-ray-solved structure (PDB 7QEH). The AMBER force field ff19SB, the monovalent ion parameters from Jung & Cheatham, and the Li/Merz ion parameters for highly charged ions were used to model the ligand and protein residues and counterions, respectively. BCC point charges of the GroP pentamer were derived with an antechamber using the program sqm. The van der Waals parameters for the phosphate ions were obtained from the phosaa10 force field. The complex between the two GroP pentamers and SlpA_amy_III was embedded in a box of OPC water molecules and minimized in three steps, where hydrogens, water molecules, and counterions, and all the system was gradually allowed to move. As an outcome, the minimized structure was printed with the closest 500 water molecules to the SlpA_amy_III/GroP pentamers complex.

## Supplementary Material

Appendix 01 (PDF)

Movie S1. **Composition of SlpA layer model based on experimental crystal structures.** This movie shows how the proposed SlpA layer model comprises experimental crystal structures. The starting point is the experimental SlpA_ac_I crystal structure, which consists of coils of SlpA_I · SlpA_I dimers connected by their N-termini. The first part of the movie shows how these SlpA_I · SlpA_I dimer chains can be uncoiled and placed on a 2D plane. The movie’s second part shows how the SlpA_I · SlpA_I dimer chains are complemented by SlpA_II and SlpA_III domains (shown in 2 SpA_motifs) and how they assemble to the complete SlpA layer.

## Data Availability

PDB, crystal structures data have been deposited in PDB (7QLE, 7QLD, 8BT9, 7QFL, 7QFG, 8ALU, 7QFI, 7QFJ, 7QFK, 8AOL, 8Q1O, 7QLH, 7QEC, and 7QEH) (73–86). All other data are included in the manuscript and/or supporting information.
