# Supplementary material for: The molecular architecture of Lactobacillus S-layer: Assembly and attachment to teichoic acids
Source: Proc Natl Acad Sci U S A. 2024 Jun 5;121(24):e2401686121. doi: 10.1073/pnas.2401686121 (PMC11181022; doi:10.1073/pnas.2401686121)
Supplement: Supplementary file 1 — Appendix 01 (PDF) [file pnas.2401686121.sapp.pdf]

PNAS

Supporting Information for
The molecular architecture of Lactobacillus S-Layer: Assembly and attachment to teichoic acids

Theo Sagmeister[1,#], Nina Gubensaek[1,#], Christoph Buhlheller[1], Christoph Grininger[1], Markus Eder[1], Anđela Đordić[1], Claudia Millán[2], Ana Medina[2], Pedro Alejandro Sánchez Murcia[3], Francesca Berni[4], Ulla Hynönen[5], Djenana Vejzovic[1], Elisabeth Damisch[1], Natalia Kulminskaya[1], Lukas Petrowitsch[1], Monika Oberer[1,6,7], Airi Palva[5], Nermina Malanović[1,6,7], Jeroen Codée[4], Walter Keller[1,6,7], Isabel Usón[2,8], Tea Pavkov-Keller[1,6,7*]

* Tea Pavkov-Keller
Email: tea.pavkov@uni-graz.at

This PDF file includes:

Supporting text
Figures S1 to S13
Tables S1 to S9
Legend for Movie S1
SI References

Other supporting materials for this manuscript include the following:

Movie S1

Supporting Information Text

Evaluation of proposed SlpA assembly with published mutagenesis data. The proposed self-assembly model is further reinforced using the results of the published mutagenesis data reported by Smit et al. 2002 [1]. These experiments quantify the effect of inserted loops between 8aa and 19aa in length on the *in vitro* ability of SlpA to assemble in crystalline form. Our assembly model proposed in this study is supported by results with mutations located in contact regions or binding sites and results with mutations located in accessible regions without close contact with other domains (Figure S9).
At the binding site between SlpA_I and its N-terminus, 3 mutation locations were tested for their effect on the S-layer assembly. These mutations are located in the SlpA binding pocket and the N-terminal tail. All 3 mutations considerably diminish or prevent the formation of crystalline assemblies, thus suggesting a crucial role of this binding site for layer assembly and confirming the assembly model presented in this study.
In the contact region between SlpA_I and SlpA_II of distinct neighboring monomers, 3 mutations were tested for their effect on the S-layer assembly. These mutations either do not assemble or show only to a lower degree of assembly. Two mutations, one in the intramolecular contact region between domain SlpA_I and SlpA_II and one near the linker between both domains, inhibit crystal layer formation. The second mutation likely affects the linker between both domains, resulting in a different domain conformation that hinders the S-layer formation. Both observations are consistent with the proposed assembly model.
Insertion mutations located at the surface of SlpA_I without close contact with other domains were compatible with layer formation; thus, these results support the proposed assembly model.

Methods

Calculation of NMR derived dissociation constants. The formula shown was used to calculate the dissociation constant Kd.

$$y = A((B + x) - \sqrt{((B + x)^2 - 4x)})$$

x … [L]/[P] ligand protein ratio
y … Δδ change of the chemical shift
A … (1/2)* Δδmax (maximal change of the chemical shift)
B = 1 + (Kd/[P])

NMR relaxation experiments. The rotational correlation time was calculated using the formula Kay et al. 1989 [2]:

Rotational correlation time

$$\tau c \approx \frac{1}{4\pi \nu \mathrm{N}} \sqrt{6\frac{T1}{T2} - 7}$$

τc…rotational correlation time [sec]
ν_N…^{15}N resonance frequency [Hz]
T_1…^{15}N T_1 relaxation time [sec]
T_2…^{15}N T_2 relaxation time [sec]

The molecular weight of a protein in solution was estimated via the rotational correlation time Cavanagh et al. 2007 [3]:

MW = τ / 0.6
τ…rotational correlation time [sec]
MW…molecular weight [kDa]

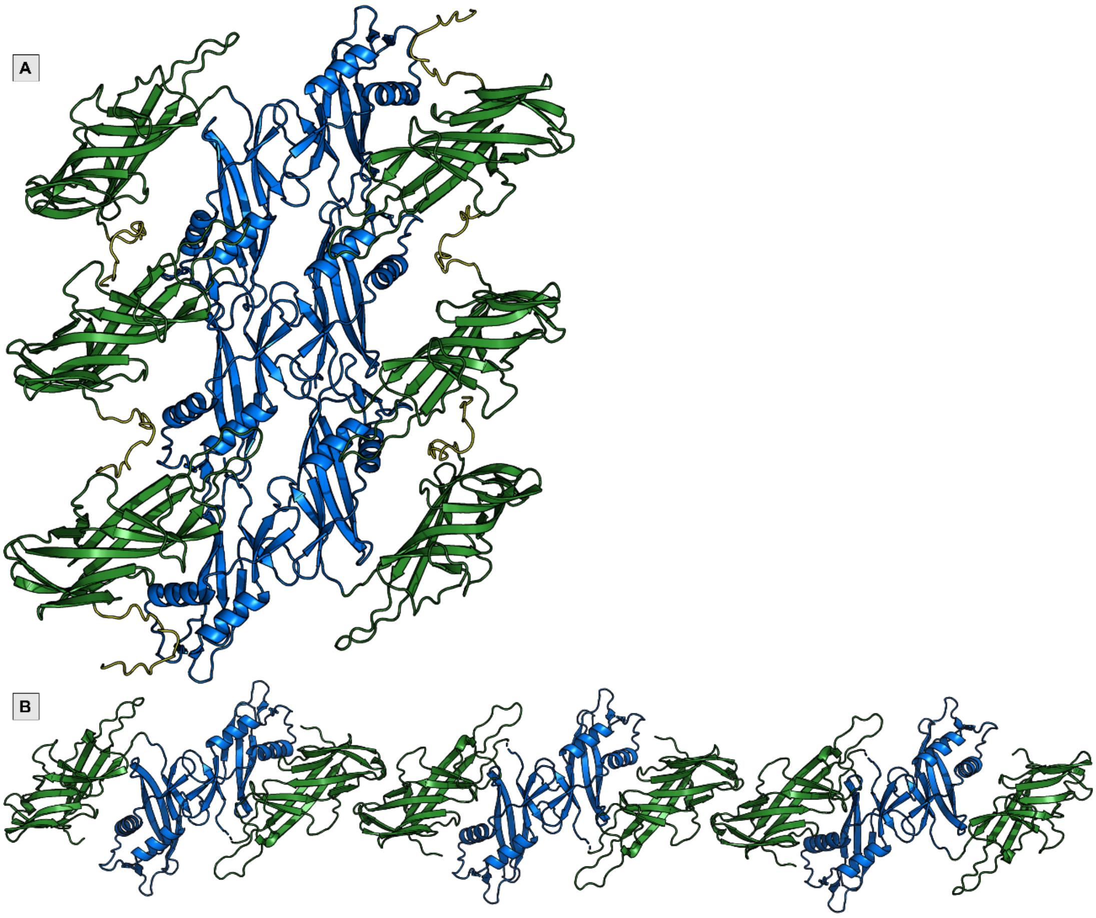

Figure S1. AlphaFold multimer prediction of full-length SlpA. A) Assembly of 6 full-length SlpA (SlpA_III facing downwards not shown) molecules as predicted by AlphaFold multimer [4,5]. The N-terminus propagates the assembly in one direction and the dimer formation of SlpA_II in the other direction. **B)** Assembly of 6 full-length SlpA (SlpA_III facing downwards not shown) molecules as predicted by AlphaFold multimer without the first 16 N-terminal residues. An alternating SlpA_I – SlpA_I and SlpA_II – SlpA_II interface is predicted with the same interactions as in the crystal structures. The angle (flexible linker) between SlpA_I and SlpA_II is adjusted and not the same as in the fully assembled model.

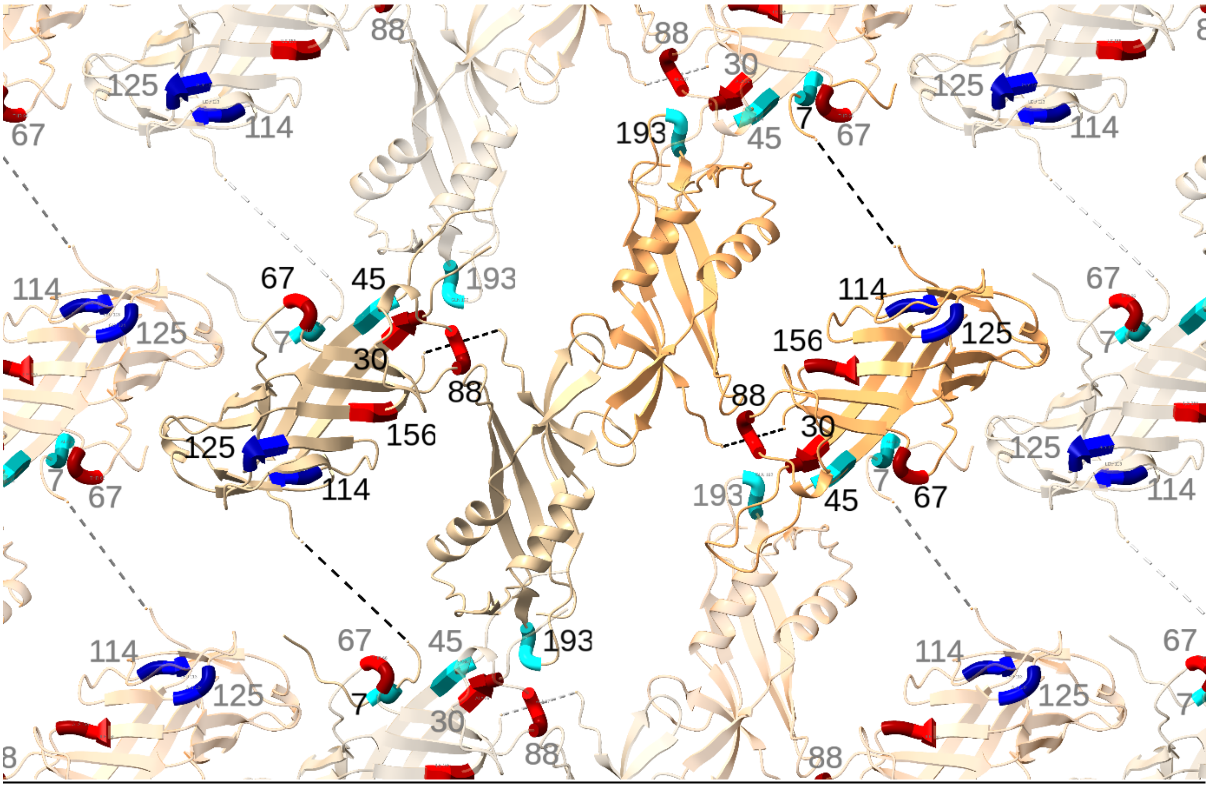

Figure S2. Self-assembly mutation data derived from Smit et al. 2002 mapped on the proposed SlpA_ac assembly model. Assembly of crystallization domains (SlpA_ac_I, SlpA_ac_II), one SlpA_ac·SlpA_ac dimer is visually highlighted compared to surrounding neighbors; linkers between domains are shown as dashed lines. The locations of mutations are colored in terms of compatibility with assembly formation according to Smit et al. 2002 [1]. Blue: mutations compatible with assembly and a high degree of assembly (25%-28%). Cyan: mutations compatible with assembly and low degree of assembly (19%-23%). Red: mutations not compatible with layer formation. The amino acid positions where the additional linker is inserted are labeled with numbers.

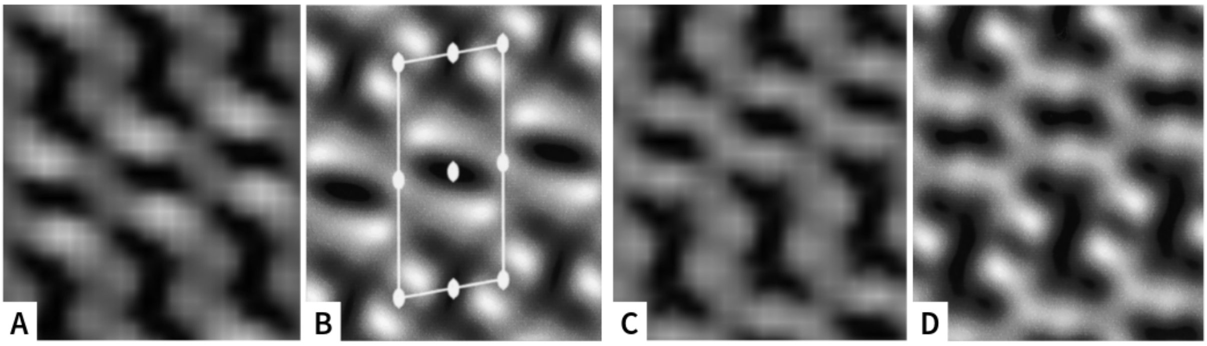

Figure S3. Comparison between simulated and available experimental projection maps. (A) simulated and (B) experimental projection maps of the full-length SlpA assembly layer. (C) simulated and (D) experimental projection maps, including only the SlpA assembly domains (SlpA_ac_**I** and SlpA_ac_**II**). Experimental projection maps are reported in Smit et al. 2001 [6]. Simulated projection maps, based on the assembly model proposed in this study, were calculated using pdb2mrc [7] in ChimeraX [8,9]

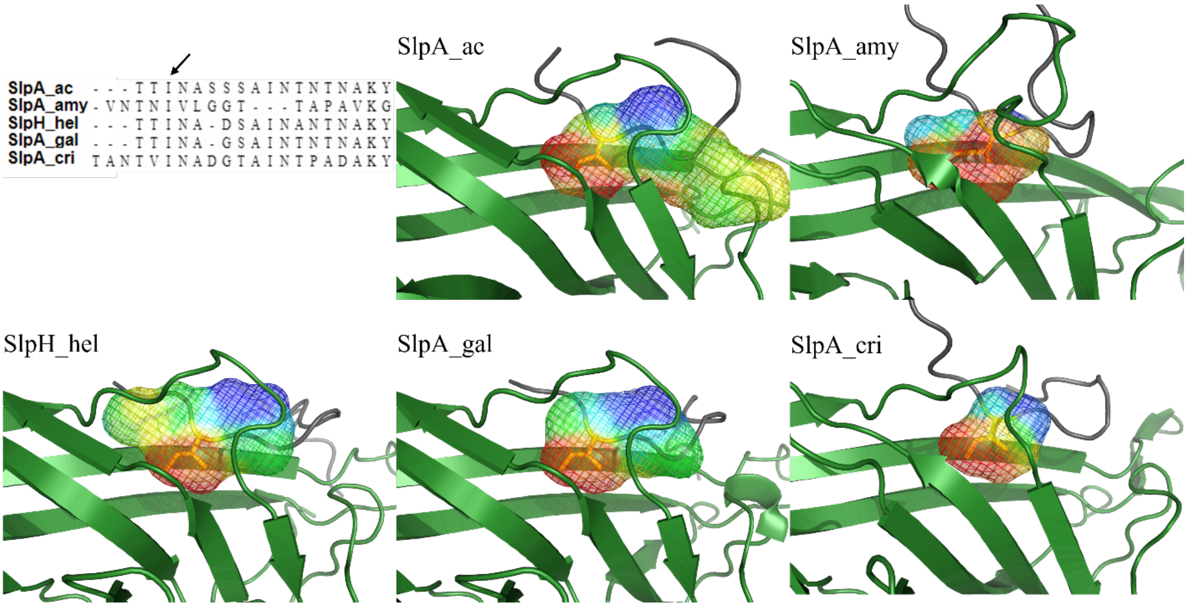

Figure S4. N-terminal cleft is highly conserved among different lactobacilli. Sequence alignment of the N-terminal residues of *L. acidophilus*, *L. amylovorus*, *L. helveticus*, *L. gallinarum,* and *L. crispastus*. Structures of SlpA_ac (7QLE) and SlpA_amy (8Q1O) are determined by X-ray crystallography, and structures of SlpH_hel, SlpA_gal, and SlpA_cri are calculated with AlphaFold multimer. The highlighted isoleucine (yellow) is conserved in all five species and positioned within the next molecule's cleft. The cleft is shown in a mesh colored from red (hydrophobic) to blue (hydrophile). Cavities were calculated using CavMan (Innophore GmbH) [10,11].

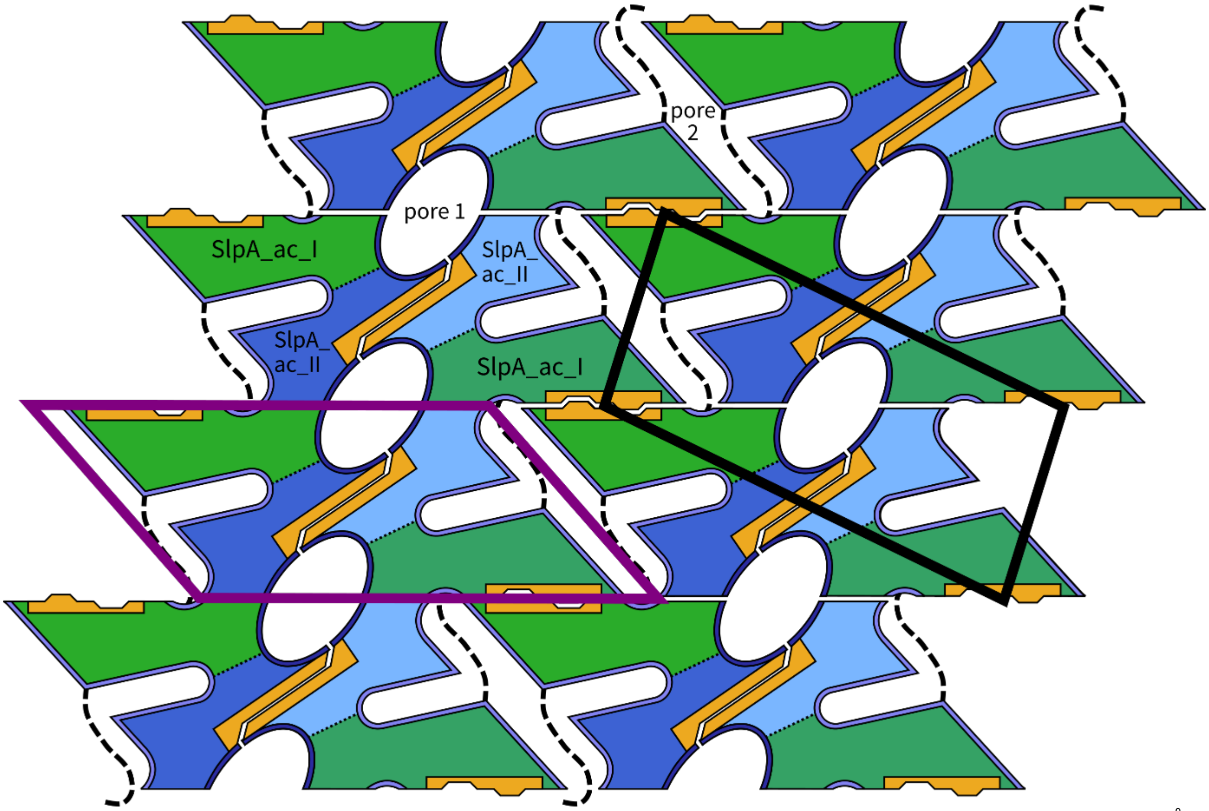

Figure S5. The primitive unit cell of the proposed SlpA assembly. Black: Unit cell with a = 118 Å, b = 53 Å, γ = 102° as reported by Smit 2001 [6] and Purple: a = 119 Å, b = 69 Å, γ = 132° as shown as one tile composed of a dimeric subunit as in Figure 4. Both tiles have the same area.

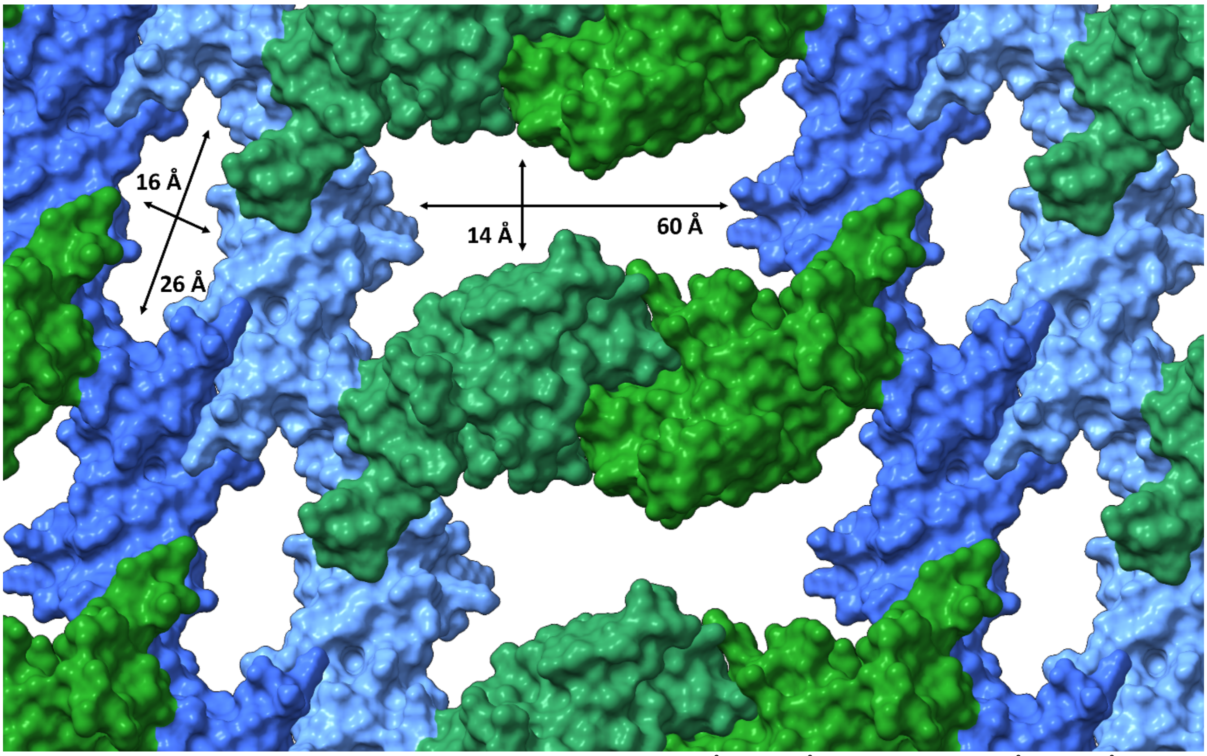

Figure S6. Pores of SlpA S-layer. A) Size of Pore 1 is ~16Å × ~26Å and Pore 2 ~14Å × ~60Å. The flexible part of the N-terminus (aa 40-49) crossing pore 2 are not shown.

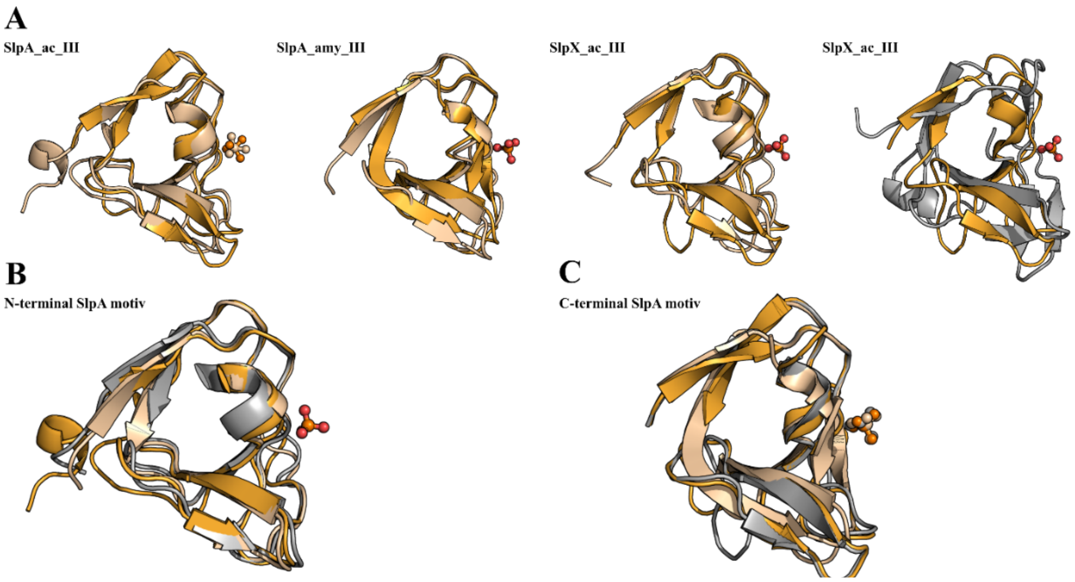

Figure S7. Conserved TAB-domain with bound phosphate. A) Alignment of the internal homodimer of N-terminal (ochre) and C-terminal (orange) SlpA motif. For SlpX_ac_III, an alignment of the additional SlpA motiv of domain II (grey) is shown. **B)** Alignment of N- and **C)** C-terminal SlpA motif of SlpA_ac_III (orange), SlpA_amy_III (ochre) and SlpX_ac_III (grey).

First N-terminal TAB-motif

Surface Layer Proteins:

```
SlpA_ac_III      ENVAEP----TVASVSKRIMHNAYYYDKDAKRVGTDSVKRYNSVSVLPNTTT-INGKTYYQVVENGKAVDKYINAANIDG
SlpA_amy_III     ANAAQTP-AAQETTKNVTIMHISTIYDKTGKATNEPALRAYDTVSVVSDPVT-INNAKFYKLAG----KDQYIKVGNVDG
SlpX_ac_III      ---------GDTNVKTYPVMVDSRAYDKNGNYLGH-MYYAYDNIDIVPTVVT-INGKTYYKVAN----KDEYVRVTNITG
P38059           PNVADP----VVPSQSKTIMHNAYFYDKDAKRVGTDKVTRYNTVTVAMNTTKLANGISYYEVIENGKATGKYINADNIDG
A0A0P0ECX7       PNVADP----VVPSQSKTIMHNAYFYDKDAKRVGTDKVTRYNTVTVAMNTTKLANGISYYEVIENGKATGKFINADNIDG
Q09FM2           PNGKDM----TVPSQSKTVMHNAFFYDKNGKRVGSDKVTRYNSATVAMNTTT-INGKAYYEVIENGKATGKFINAANIDG
```

Non-Surface Layer Proteins:

```
A0A809KBB        -NNSSSAATPSEPETSGYVMKKAYIYNKKGERQSG-YYAAYYGIKYYGSTVTLDNGKTALKVGD-----DRYVMASNVLG
I7LAA9           NNNATTNSDQAAVPIIKYLYHNAYLYDQAGKRTNTL-VLKRFNFINTYGKLT-IAGKKYYRIDK-----AHYVAANNIDP
A0A4V6RD31       PAAPSSEAPKGATKVEKNLMHNAYVYNEKGERIKDIKLLQEDNVIPTYGTKT-IKNKKYYRIGE-----NQYIAAGNIDG
A0A6P1TWR6       ----ANI-CSCQDTITKTLMHAAYLYDNQGVRIKKPFIKAYTAVVVAKTPVV-INGIKYYKVPC----KDEYIKSGNFDG
                                   :   :  *:: .                         .    .:        .::   *.
```

Second C-terminal TAB-motif

Surface Layer Proteins:

```
SlpA_ac_III_c    TKRTLKHNAYVYASSKKRA----NKVVLKKGEVVTTYGASYTFKNGQKYYKIGD--NTDKTYVKVANFR-
SlpA_amy_III_c   TSRTLKHNSYVYKSSGKRA----NKKTLKKGSSVTTYGKSFMIA-GHQMYRIGK--N---QYVKKANF--
SlpX_ac_III_c    NQRTLKHNAYIYWSSYRRTPG---TGKMYRGQTVTTYGPQMKFKNGKKYYRIEGCRNNNKRYIKAVNFY-
P38059_c         TKRTLKHNAYVYKTSKKRA----NKVVLKKGTEVTTYGGSYKFKNGQRYYKIGA--NTEKTYVKVANFE-
A0A0P0ECX7_c     TKRTLKHNAYVYKTSKKRA----NKVVLKKGTEVTTYGDSYKFKNGKKYYKIGA--DTKKTYVNAANF--
Q09FM2_c         TKRTLKHNAYVYKSSKKRA----NKVVLKKGTEVVTYGGAYTFKNGKQYYKIGN--NTDKTYVKVSNF—
```

Non-Surface Layer Proteins:

```
A0A809KBB_c      NSRVMKHNAYIYNHSGRRA----NWRVLRKGTPIKTYGSKFNIN-GKSYYRIGK--G---LYVKAANF--
I7LAA9_c         IKRTLKRNSFIYDEFGKLVKK--TTNATKKGRKINTYGSIVKIN-GKKFYTVGK--N---QFIKAINFKK
A0A4V6RD31_c     TMKVLKHNAYVYNQYANRD----NDLKYKKGQAVNTYGSAVKIL-GKKYYKVGV--H---EYVKKANF--
A0A6P1TWR6_c     TSRVLRHNAFVYNHKGKAVRIKGIKRLLKKWRLVRTYGAPFNIN-GHQMYRIAK--N---RYIKVKNFK-
                   :.:::*:::*    .            :   : ***    :  *:  * :         :::  **
```

Figure S8. Conserved TAB-domain motif across different proteins in *Lactobacillus.* P38059, *L. helveticus*; A0A0PECX7L, *L. gallinarum*; Q09FM2 *L. crispatus.* Residues highlighted in yellow are conserved and involved in TAB-domain binding motif and correspond to the residues shown in Figure 5. Residues indicated with a star show only backbone interaction. The asterisk (*), colon (:), and semicolon (.) indicate fully conserved residue, conserved between groups of strongly similar properties and conserved between weakly similar properties, respectively.

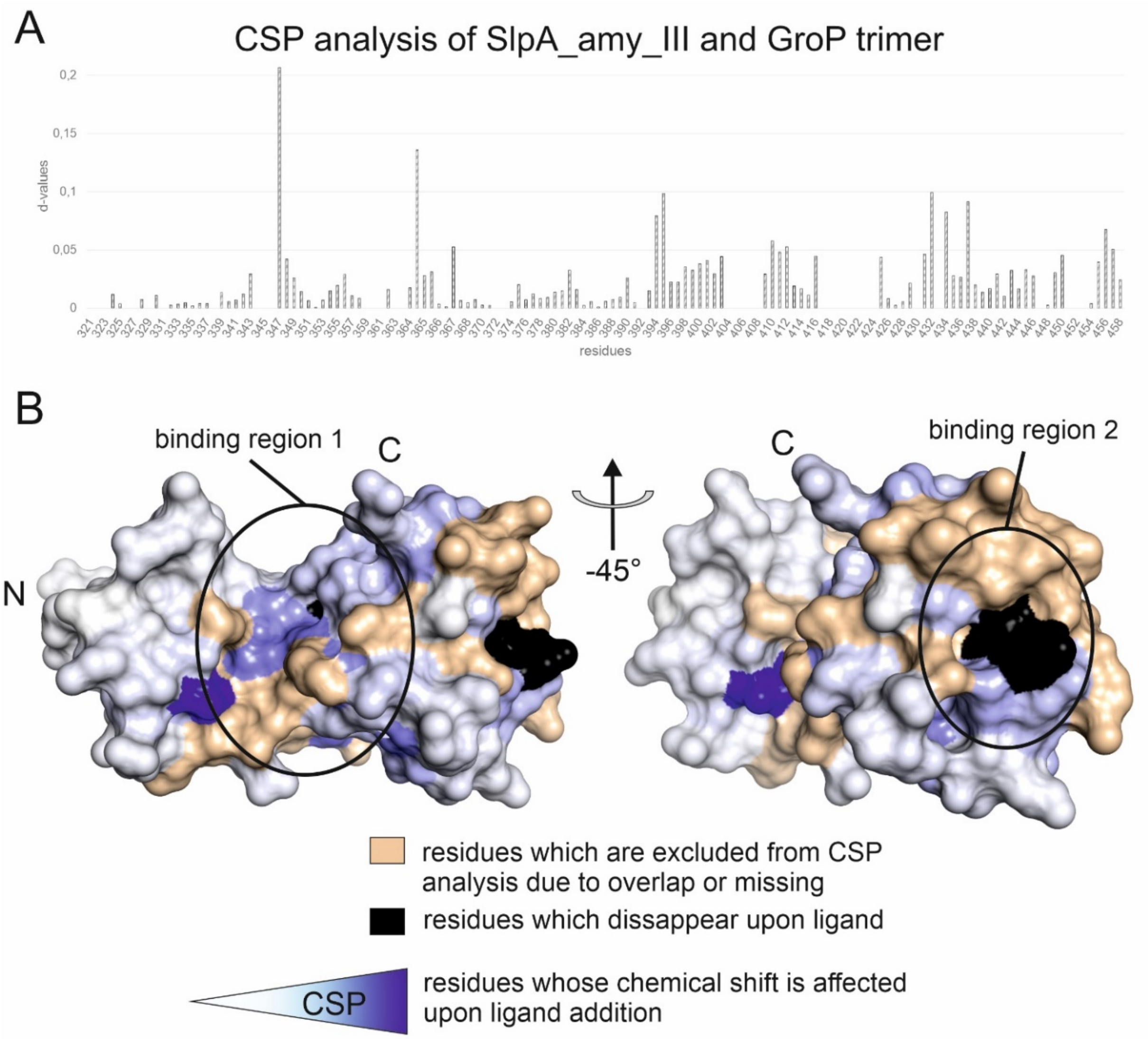

Figure S9. CSP analysis of SlpA_amy_III and GroP trimer. A: The presented graph shows the calculated d-values for each residue. Residues with high d-values are highly influenced by ligand interaction and are either located in or close to the binding pocket or experience conformational changes.

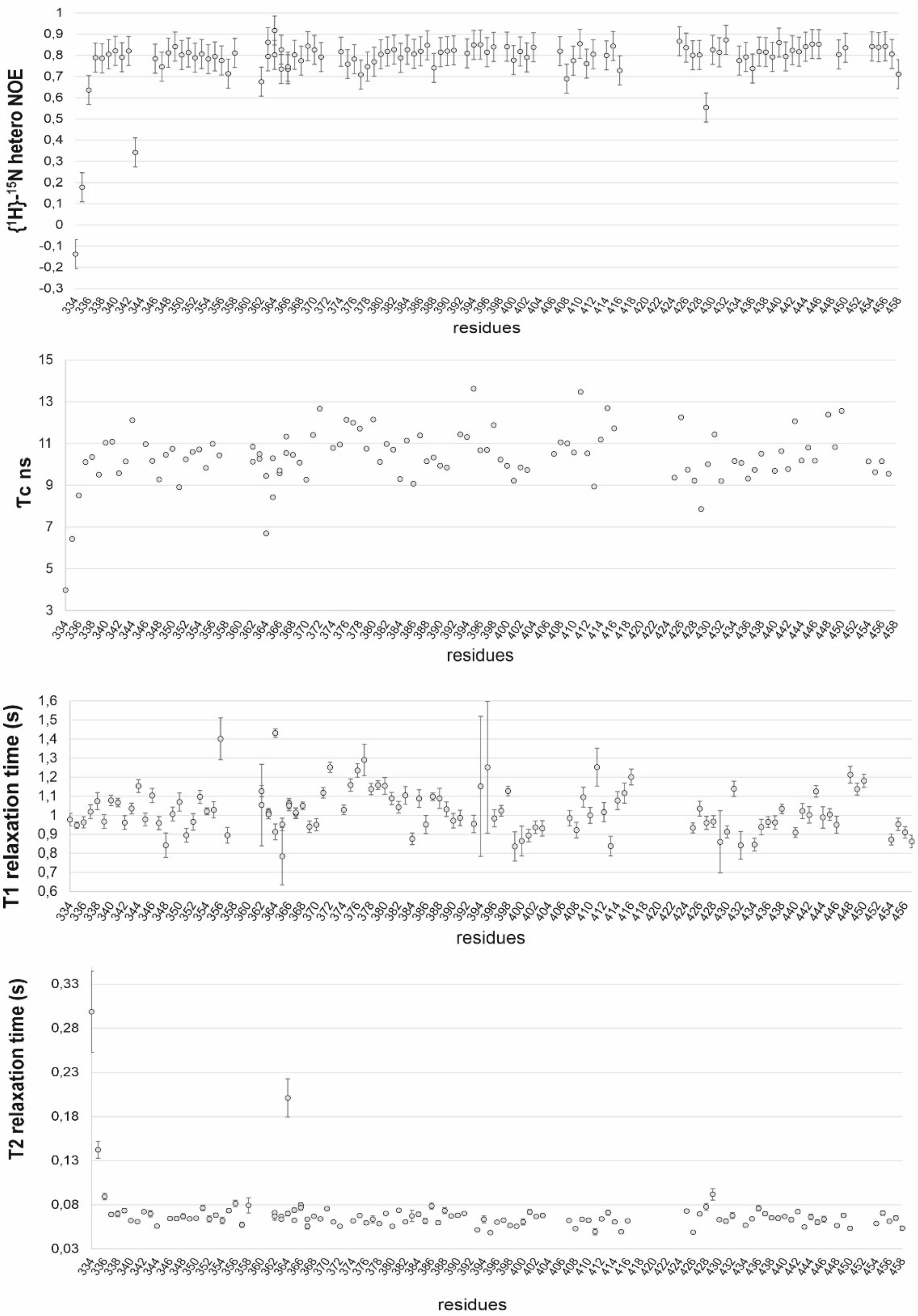

Figure S10. Analysis of SlpA_amy_III dynamics via NMR relaxation experiments. For each residue {^{1}H}-^{15}N- hetero NOE values and rotational correlation coefficients were determined. T1 and T2 relaxation data were used for the calculation of rotational correlation coefficients. Data reveals increased dynamics only at the N-terminal region, whereas the rest of the protein exhibits high {^{1}H}-^{15}N- hetero NOE values indicating a rigid stable fold of SlpA_amy_III. The average rotational correlation time of SlpA_amy_III in solution is 10 nsec corresponding to a well-structured 21 kDa protein (see ‘Methods’). The SlpA_amy_II construct has an MW of approximately 16 kDa.

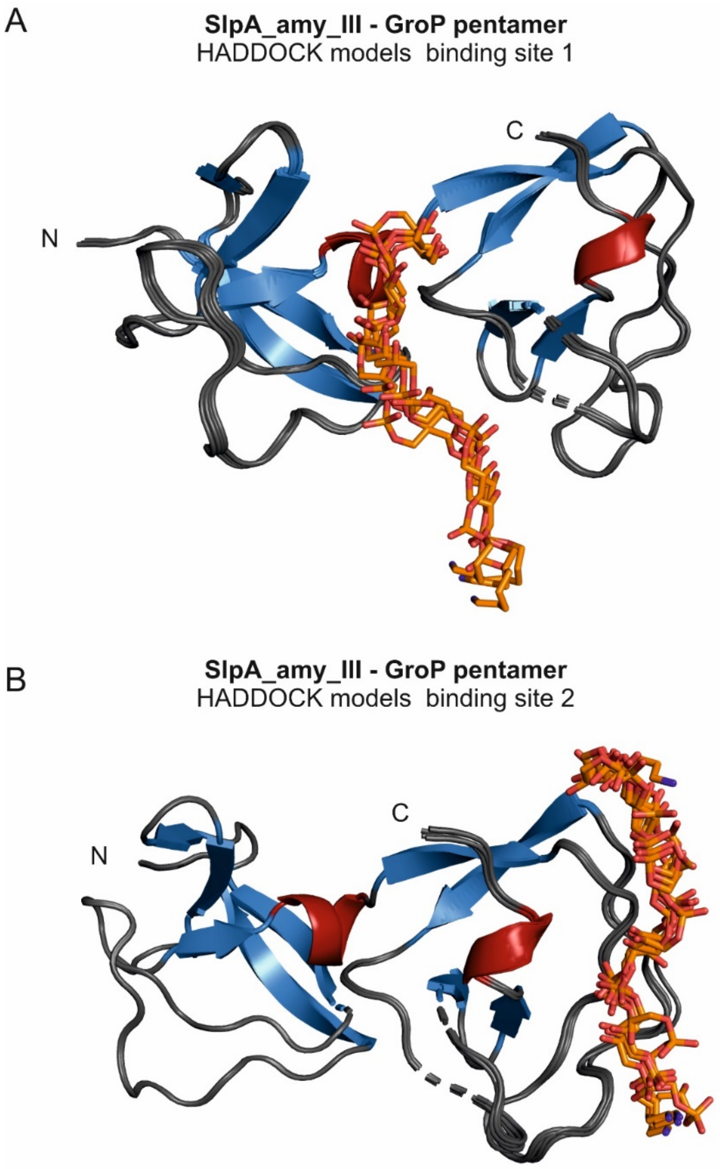

Figure S11. CSP derived HADDOCK models of SlpA_amy_II and GroP pentamer. For each binding site a separate HADDOCK run was performed. Models of lowest HADDOCK-scored clusters for binding site 1 are shown in (A), for binding site 2 in (B).

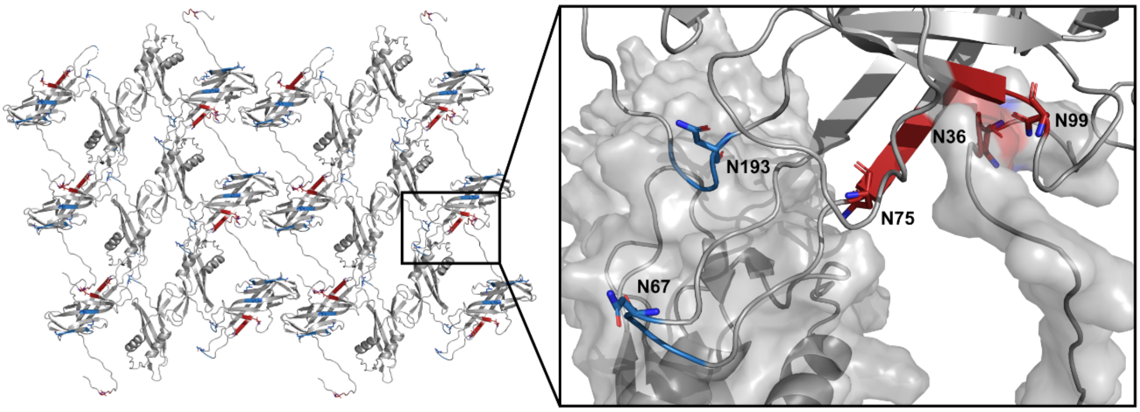


```
         40         50         60         70         80         90        100        110        120        130        140        150
 ATTINASSS AINTNTNAKY DVDVTPSVSA VAANTANNTP AIAGNLTGTI SASYNGKTYT ANLKADTENA TITAAGSTTA VKPAELAAGV AYTVTVNDVS FNFGSENAGK TVTLGSANSN
        160        170        180        190        200        210        220        230        240        250        260        270
VKFTGTNSDN QTETNVSTLK VKLDQNGVAS LTNVSIANVY AINTTDNSNV NFYDVTSGAT VTNGAVSVNA DNQGQVNVAN VVAAINSKYF AAQYADKKLN TRTANTEDAI KAALKDQKID
        280        290        300        310        320        330        340        350        360        370        380        390
VNSVGYFKAP HTFTVNVKAT SNTNGKSATL PVVVTVPNVA EPTVASVSKR IMHNAYYYDK DAKRVGTDSV KRYNSVSVLP NTTTINGKTY YQVVENGKAV DKYINAANID GTKRTLKHNA
        400        410        420        430        440
YVYASSKKRA NKVVLKKGEV VTTYGASYTF KNGQKYYKIG DNTDKTYVKV ANFR
```

Figure S12. Putative N-glycosylation sites (Asn - X - Ser/Thr) of SlpA_ac. The possible N-glycosylation sites of SlpA_ac are depicted in red/blue with the sticks representation for the respective asparagines. Sites near the interaction surface between SlpA_ac monomers are shown in detail and colored in red, surface exposed sites are colored blue. The sequence of SlpA_ac is shown with the highlighted glycosylation sites.

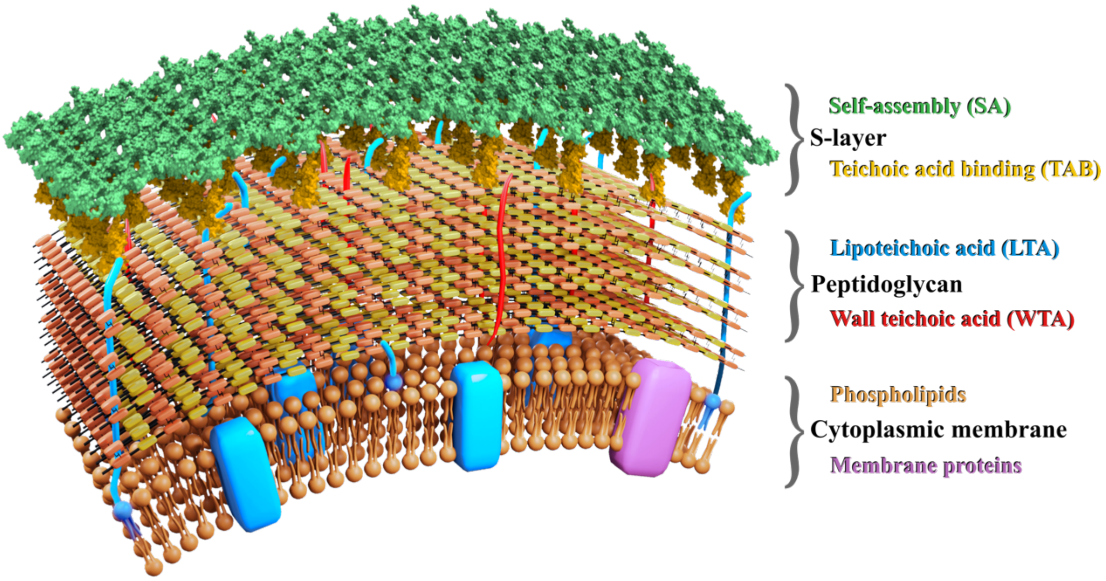

Figure S13. Schematical overview of the cell wall composition of *Lactobacillus*. S-layer composed of SlpA is shown as the outermost layer. The domains involved in the self-assembly are shown in green and the TAB-domain is shown in yellow. Putative glycosylation of the S-layer is omitted in this figure. Lipoteichoic acids anchored in the cytoplasmic membrane are shown in blue and peptidoglycon-anchored wall teichoic acids are in red. Membrane proteins are colored in violet and in light blue.

Table S1. List of to date available experimental structures of assembled S-layers and SLP fragments.

Organism	Protein / Accession	PDB Code
**Gram-positive**		
Lactobacillus acidophilus	SlpA (P35829)	7QLD, 7QFL, 7QLE, 7QFG, 8ALU, 8BT9
Lactobacillus acidophilus	SlpX (Q5FLN0)	7QFI, 7QFJ, 7QFK,8AOL
Lactobacillus amylovorus	SlpA (E4SK47)	7QLH, 7QEC, 7QEH, 8Q1O
Geobacillus stearothermophilus	SbsB (Q45664)	4AQ1 [12]
Geobacillus stearothermophilus	SbsC (O68840)	2RAI, 4UIC, 4UID, 4UIE, 4UJ6, 4UJ7, 4UJ8, 5FTX, 5FTY [13–16]
Bacillus anthracis	Sap (P49051)	3PYW, 6BT4, 6QX4, 6HHU [17–19]
Bacillus anthracis	EA1 (P94217)	8OPR [20]
Clostridium difficile	SlpA (Q183M8)	3CVZ, 7ACV, 7ACW, 7ACX, 7ACY, 7ACZ, 7QGQ, 8BBY [21,22]
**Gram-negative**		
Deinococcus radiodurans	SlpA (Q9RRB6)	7ZGX, 7ZGY, 8AE1, 8ACQ, 8AGD [21,22]
Deinococcus radiodurans	HPI (P56867)	8CKA [23]
Caulobacter cresentus (Caulobacter vibrioides)	RsaA (P35828)	5N8P, 6P5T, 6T72, 6Z7P, 7PEO, 8BQE, 5N97 [24–27]
**Archaea**		
Haloferax volcanii (Halobacterium volcanii)	csg (P25062)	7PTP, 7PTR, 7PTT, 7PTU [28]
Methanosarcina mazei	ORF492 (Q50245)	1L0Q [29]
Methanosarcina acetivorans	Major S-layer protein (Q8TSG7)	3U2H, 3U2G [30]
Sulfolobus acidocaldarius	SlaA (Q4J6E5)	8AN2, 8AN3, 7ZCX, 8QP0 [31]
Sulfolobus acidocaldarius	SlaA/SlaB (Q4J6E6)	8QOX [31]

Table S2. Constructs used in this work. Residue numbering includes signal sequence.

Construct	Vector	Residues
SlpA_ac_I	pJC40	32-198
SlpA_ac_I S115C	pJC40	32-198
SlpA_ac_II	pJC40	199-308
SlpA_ac-III	pET-28a(+)-TEV	309-444
SlpA_ac_III PO4	pET-28b	309-444
SlpX_ac_I	pJC40	31-230
SlpX_ac_II	pJC40	194-362
SlpX_ac_II S133C	pET-28a(+)-TEV	194-362
SlpX_ac_III	pET-28b	363-499
SlpA_amy_I	pJC40	31-213
SlpA_amy I_ΔN18	pJC40	48-213
SlpA_amy_II	pET-28a(+)-TEV	213-326
SlpA_amy_III	pET-28b	322-456

Table S3. Primers used for cloning. All primers were either designed with the NEBaseChanger, SerialCloner, or by hand.

Primer	Sequence
SlpA_ac I R	ATGACGACCTTCGATATG
SlpA_ac I R pet28	tatatctcgagACTGTTATCAGTAGTGTTAATTGCGTATACG
SlpA_ac I F pet28	tatatccatgggcGCTACTACTATTAACGCAAG
SlpA_ac I F d17N pet28	tatatccatgggcAAGTACGATGTTGATGTAA
SlpA_ac III F	ATCCATGGAAAATGTTGCTGAG
SlpA_ac III R	ATCTCGAGTCTAAAGTTTGCAACCTTAA
SlpA_amy III F	ATCCATGGTTTCAGTTGCAAAC
SlpA_amy III R	ATCTCGAGGAAGTTTGCCTTCTTTA
SlpX_ac I F	ATCATATGGATACCGCAGTGAATGTTGGTAG
SlpX_ac I R	ATGGATCCTCAATTGCTATTGGTAAAATTCAGCG
SlpX_ac II F	tatatccatgggcACCGATACCACACAGAATCC
SlpX_ac II R	tatatctcgagGCTGGTTTTCACCCAGATGT
SlpX_ac III F	ATCCATGGCGGCACTGGTTAAACCGGCA
SlpX_ac III R	CATGGTATATCTCCTTCTTAAAG
SlpX_ac II F S133C	TAATGTGCTGtGCAATAGCAC
SlpX_ac II R S133C	TTGCCATAGATGCTATAG
SlpA_ac I R S115C	ACAGTCTTACCTGCATTTTC
SlpA_ac I F S115C	TACCCTTGGTtgcGCTAACTCAAATG
SlpA_amy_I F	GCCATATGGACGTTAACACTAACATTG
SlpA_amy_I R	GCGGATCCTTAGCTCTTTGCA
SlpA_amy_I_ΔN18 F	tatatccatgggcAAAGGCGATGTTAATGTAAC
SlpA_amy_I_ΔN18 R	atatggatcctcagtgatgatggtgatgatgGCTCTTTGCATTAGTTACAT

Table S4. Screens and final conditions used for crystallization of all fragments.

**Protein**	**7QLE: SlpA_ac_I**	**7QLD: SlpA_ac_I S115C**	**8BT9: SlpA_ac_II_dimer**
Condition	A5, JCSG-plusTM HT-96 Eco screen, Molecular Dimensions; 0.2 M Magnesium formate dihydrate, 20 % w/v PEG 3350	D5, JCSG-plusTM HT-96 Eco screen, Molecular Dimensions; 0.1 M HEPES pH 7.5, 70 % v/v MPD	F4, SG1 Screen HT-96, Molecular Dimensions; 1.0 M Sodium citrate tribasic dihydrate, 0.1 M MES, pH 6.5
**Protein**	**7QFL: SlpA_ac_II**	**7QFG: SlpA_ac_III**	**8ALU: SlpA_ac_III PO4**
Condition	A1, JCSG-plusTM HT-96 Eco screen, Molecular Dimensions; 0.18 M Lithium sulfate, 90 mM Sodium acetate pH 4.5, 45 % w/v PEG 400	A9, Index HR2-144, Hampton; 0.1 M BIS-TRIS pH 5.5, 3.0 M Sodium chloride	G8, Index HR2-144. Hampton; 0.2 M Ammonium acetate, 0.1 M HEPES pH 7.5, 25% w/v Polyethylene glycol 3350
**Protein**	**7QFI: SlpX_ac_I**	**7QFJ: SlpX_ac_II**	**7QFK: SlpX_ac_II S133C**
Condition	E2, PGA HT-96 Eco Screen, Molecular Dimensions;0.2 M Potassium bromide, 0.2 M Potassium thiocyanate, 0.1 M MES pH 6.5, 3 % w/v γ-PGA, 20 % v/v PEG 500 MME	C7, MIDASplus™ MD1–106, Molecualr Dimensions; 0.1 M Tris pH 8, 50 % v/v Pentaerythritol propoxylate (5/4 PO/OH)	H12, Index HR2-144, Hampton; 0.15 M Potassium bromide, 30% w/v Polyethylene glycol monomethyl ether 2,000
**Protein**	**8AOL: SlpX_ac_III**	**8Q1O: SlpA_amy_I**	**7QLH: SlpA_amy_I ΔN18**
Condition	F6, Wizard Classic 1 and 2, Molecular Dimensions; 20% (w/v) PEG 3000, 100 mM Tris base/ Hydrochloric acid pH 7.0, 200 mM Calcium acetate	A5, PGA HT-96 Eco Screen, Molecular Dimensions; 1.0 M Ammonium formate, 0.1 M Sodium acetate pH 5.0, 8 % w/v γ-PGA	B5, JCSG-plusTM HT-96 Eco screen, Molecular Dimensions; 40% MPD, 5% PEG8000, 100 mM MES pH 6.5
**Protein**	**7QEC: SlpA_amy_II**	**7QEH: SlpA_amy_III**	
Condition	B12, Morpheus® HT-96, Molecular Dimensions; 12.5% w/v PEG 1000, 12.5% w/v PEG, 3350, 12.5% v/v MPD, 0.09 M Halogens, 0.1 M Bicine/Trizma Base pH 8.5	G5, Morpheus® HT-96, Molecular Dimensions; 1.0M pH 7.5 Sodium HEPES; MOPS, 40 v/v PEG 500; 20% w/v PEG 20000, 0.2M Sodium formate; 0.2M Ammonium acetate; 0.2M Sodium citrate tribasic dihydrate; 0.2M Potassium sodium tartrate tetrahydrate; 0.2M Sodium oxamate	

Table S5: Crystallographic table for data collection and refinement statistics. Values in parentheses are for the highest-resolution shell.

	SlpA_ac_I	SlpA_ac_I S115C	SlpA_ac_II Dimer	SlpA_ac_II	SlpA_ac_III
**Data collection**					
PDB Code	7QLE	7QLD	8BT9	7QFL	7QFG
Structure solution	MR with 7QLD	SAD with Hg	MR with 7QFL	MR with 7QEC	MR with 8ALU
Wavelength (Å)	0.978	0.976	1.033	1.033	1.254
Space group	$C\,2$	$P\,6_522$	$P\,3_221$	$P\,3_121$	$C\,2$
Cell dimensions					
a, b, c (Å)	130.12, 39.03, 60.05	85.36, 85.36, 191.96	137.69, 137.69, 51.30	55.85, 55.85, 63.73	100.62, 43.94, 42.48
α, β, γ (°)	90, 101.66, 90	90, 90, 120	90, 90, 120	90, 90, 120	90, 98.82, 90
Resolution (Å)	48.37-2.6 (2.693-2.6)	48.38-2.153 (2.23-2.153)	45.07-2.1 (2.175-2.1)	48.37-1.4 (1.45-1.4)	41.99-1.65 (1.709-1.65)
R_{merge}	0.08334 (0.3661)	0.01473 (0.2701)	0.01155 (0.4849)	0.0245 (0.2546)	0.0524 (0.1046)
I / σI	7.36 (2.07)	26.84 (2.73)	23.76 (1.57)	14.11 (2.66)	11.45 (6.41)
Completeness (%)	99.08 (99.11)	99.80 (99.07)	99.97 (99.97)	99.96 (99.74)	99.32 (99.37)
Redundancy	1.9 (1.9)	2.0 (2.0)	2.0 (2.0)	2.0 (2.0)	1.8 (1.8)
CC½	0.99 (0.722)	1 (0.92)	1 (0.703)	0.998 (0.862)	0.989 (0.958)
**Refinement**					
No. reflections	9250 (894)	23206 (2240)	32852 (3272)	23130 (2265)	22100 (2193)
R_{work}	0.2032 (0.2966)	0.2288 (0.3629)	0.1892 (0.3184)	0.1719 (0.2590)	0.1591 (0.1610)
R_{free}	0.2493 (0.3567)	0.2799 (0.3859)	0.2221 (0.2975)	0.2013 (0.2750)	0.1797 (0.2019)
No. atoms	2322	2325	2691	884	1303
Protein	2193	2251	2529	790	1088
Ligand/ion	0	2	9	12	37
Water	129	72	153	85	197
B-factors	34.95	71.19	76.60	20.97	17.96
Protein	35.52	71.39	76.99	19.71	16.21
Ligand/ion		100.46	47.95	25.97	22.66
Water	25.25	64.19	71.81	32.24	27.18
R.m.s. deviations					
Bond lengths (Å)	0.014	0.014	0.010	0.017	0.015
Bond angles (°)	2.04	2.02	1.66	2.02	1.9
Ramachandran					
Favored (%)	98.66	97.71	98.45	98.99	97.76
Allowed (%)	1.34	2.29	1.55	1.01	2.24
Outliers (%)	0	0	0	0	0
Rotamer outliers (%)	2.07	3.64	2.5	0	0
Clashscore	1.84	2.24	5.36	1.0	0.9

	SlpA_ac_III PO_4	SlpX_ac_I	SlpX_ac_II	SlpX_ac_II S133C	SlpX_ac_III
**Data collection**					
PDB Code	8ALU	7QFI	7QFJ	7QFK	8AOL
Structure solution	MR with 7QEH	ARCIMBOLDO Lite	MR with 7QFK	MR with RoseTTAFold	MR with 8ALU
Wavelength (Å)	0.950	0.873	0.942	0.965	1.0332
Space group	$C\ 2$	$P\ 2_1$	$P\ 2_1$	$P\ 2_1 2_1 2_1$	$P\ 6_2 22$
Cell dimensions					
a, b, c (Å)	80.43, 27.05, 61.89	37.35, 38.34, 82.74	65.67, 143.01, 73.44	63.16, 98.87, 155.13	133.17, 133.17, 70.57
α, β, γ (°)	90, 91.04, 90	90, 93.92, 90	90, 96.69, 90	90, 90, 90	90, 90, 120
Resolution (Å)	33.44-2.094 (2.169-2.094)	41.28-1.5 (1.554-1.5)	46.03-2.5 (2.589-2.5)	48.98-2.484 (2.573-2.484)	44.65-2.4 (2.486-2.4)
R_{merge}	0.04635 (0.5546)	0.05216 (0.3983)	0.03645 (0.2614)	0.03315 (0.2435)	0.03782 (0.305)
$I / \sigma I$	15.46 (1.53)	7.81 (1.84)	17.32 (3.11)	13.20 (2.77)	10.71 (2.06)
Completeness (%)	84.51 (43.04)	98.70 (97.16)	99.88 (99.94)	99.58 (96.36)	99.93 (99.86)
Redundancy	2.9 (2.3)	1.9 (1.9)	2.0 (2.0)	2.0 (2.0)	2.0 (2.0)
CC½	0.999 (0.751)	0.996 (0.735)	0.998 (0.871)	0.999 (0.865)	0.998 (0.866)
**Refinement**					
No. reflections	6868 (340)	37258 (3630)	46452 (4623)	34914 (3313)	14914 (1442)
R_{work}	0.2079 (0.3270)	0.1900 (0.2937)	0.2051 (0.3112)	0.2012 (0.3001)	0.1787 (0.3439)
R_{free}	0.2597 (0.3582)	0.2278 (0.3221)	0.2318 (0.3582)	0.2351 (0.3920)	0.2045 (0.3047)
No. atoms	1100	2245	8004	5566	1316
Protein	1045	2012	7788	5253	1143
Ligand/ion	10	1	0	38	29
Water	45	232	216	275	144
B-factors	39.63	20.94	49.4	46.71	49.94
Protein	39.48	19.8	49.72	46.93	49.77
Ligand/ion	51.26	22.99		59.14	55.68
Water	40.47	30.76	37.72	40.8	50.10
R.m.s. deviations					
Bond lengths (Å)	0.002	0.015	0.014	0.014	0.015
Bond angles (°)	0.41	1.85	1.88	1.95	2.02
Ramachandran					
Favored (%)	95.35	98.02	98.33	97.39	95.52
Allowed (%)	4.65	1.98	1.67	2.61	4.48
Outliers (%)	0	0	0	0	0
Rotamer outliers (%)	0	0	1.11	1.64	1.65
Clashscore	2.36	1.5	1.84	3.0	2.18

	SlpA_amy_I	SlpA_amy_I ΔN18	SlpA_amy_II	SlpA_amy_III
**Data collection**				
PDB Code	8Q1O	7QLH	7QEC	7QEH
Structure solution	MR with 7QLH	MR with 7QLE	ARCIMBOLDO Lite	SAD with Se-Methionine
Wavelength (Å)	1.004	0.942	0.999	0.978
Space group	*C* 2	$P\ 2_12_12$	$P\ 4_12_12$	$P\ 2_12_12_1$
Cell dimensions				
a, *b*, *c* (Å)	133.19, 43.85, 86.38	49.54, 91.75, 93.61	38.63, 38.63, 142.44	30.56, 43.48, 89.10
α, β, γ (°)	90, 95.49, 90	90, 90, 90	90, 90, 90	90, 90, 90
Resolution (Å)	41.64-3.401 (3.522-3.401)	43.79-2.3 (2.382-2.3)	35.61-1.951 (2.021-1.951)	39.08-1.675 (1.734-1.675)
R_{merge}	0.1963 (0.7629)	0.05662 (0.4732)	0.1144 (0.94)	0.1088 (0.9939)
$I / \sigma I$	5.53 (1.71)	19.32 (4.17)	17.79 (2.42)	19.84 (3.76)
Completeness (%)	97.31 (98.15)	99.02 (99.11)	98.14 (81.32)	99.39 (94.27)
Redundancy	3.3 (3.3)	6.3 (6.5)	18.9 (13.2)	12.2 (10.5)
CC½	0.989 (0.789)	0.999 (0.959)	0.999 (0.759)	0.999 (0.811)
**Refinement**				
No. reflections	7029 (690)	19426 (1901)	8344 (666)	14203 (1315)
R_{work}	0.2506 (0.4206)	0.2228 (0.2570)	0.1846 (0.2311)	0.1626 (0.2374)
R_{free}	0.3306 (0.4880)	0.2829 (0.3345)	0.2445 (0.2618)	0.1905 (0.2363)
No. atoms	2702	2530	821	1053
Protein	2676	2458	776	973
Ligand/ion	10	11	0	5
Water	16	61	45	75
B-factors	75.72	55.81	37.76	24.47
Protein	75.82	55.97	37.47	23.89
Ligand/ion	119.97	51.26		32.24
Water	32.30	50.13	42.76	31.46
R.m.s. deviations				
Bond lengths (Å)	0.007	0.003	0.008	0.009
Bond angles (°)	1.50	0.55	0.78	1.00
Ramachandran				
Favored (%)	92.61	96.55	100	100.00
Allowed (%)	7.10	3.45	0	0
Outliers (%)	0.28	0.00	0	0
Rotamer outliers (%)	1.67	0.36	1.2	1.87
Clashscore	3.40	3.0	3.24	0.5

Table S6. Sequences used for AlphaFold multimer calculations

Structure	Input sequences
Dimer SlpA·SlpA	>SlpA<br>ATTINASSSAINTNTNAKYDVDVTPSVSAVAANTANNTPAIAGNLTGTISASYNGKTYTANL<br>KADTENATITAAGSTTAVKPAELAAGVAYTVTVNDVSFNFGSENAGKTVTLGSANSNVKFT<br>GTNSDNQTETNVSTLKVKLDQNGVASLTNVSIANVYAINTTDNSNVNFYDVTSGATVTNGA<br>VSVNADNQGQVNVANVVAAINSKYFAAQYADKKLNTRTANTEDAIKAALKDQKIDVNSVG<br>YFKAPHTFTVNVKATSNTNGKSATLPVVVTVPNVAEPTVASVSKRIMHNAYYYDKDAKRV<br>GTDSVKRYNSVSVLPNTTTINGKAYYQVVENGKAVDKYINAANIDGTKRTLKHNAYVYASS<br>KKRANKVVLKKGEVVTTYGASYTFKNGQKYYKIGDNTDKTYVKVANFR<br>>SlpA<br>ATTINASSSAINTNTNAKYDVDVTPSVSAVAANTANNTPAIAGNLTGTISASYNGKTYTANL<br>KADTENATITAAGSTTAVKPAELAAGVAYTVTVNDVSFNFGSENAGKTVTLGSANSNVKFT<br>GTNSDNQTETNVSTLKVKLDQNGVASLTNVSIANVYAINTTDNSNVNFYDVTSGATVTNGA<br>VSVNADNQGQVNVANVVAAINSKYFAAQYADKKLNTRTANTEDAIKAALKDQKIDVNSVG<br>YFKAPHTFTVNVKATSNTNGKSATLPVVVTVPNVAEPTVASVSKRIMHNAYYYDKDAKRV<br>GTDSVKRYNSVSVLPNTTTINGKAYYQVVENGKAVDKYINAANIDGTKRTLKHNAYVYASS<br>KKRANKVVLKKGEVVTTYGASYTFKNGQKYYKIGDNTDKTYVKVANFR
Dimer SlpA1·SlpA1	>SlpA1<br>ATTINASSSAINTNTNAKYDVDVTPSVSAVAANTANNTPAIAGNLTGTISASYNGKTYTANL<br>KADTENATITAAGSTTAVKPAELAAGVAYTVTVNDVSFNFGSENAGKTVTLGSANSNVKFT<br>GTNSDNQTETNVSTLKVKLDQNGVASLTNVSIANVYAINTTDNS<br>>SlpA1<br>ATTINASSSAINTNTNAKYDVDVTPSVSAVAANTANNTPAIAGNLTGTISASYNGKTYTANL<br>KADTENATITAAGSTTAVKPAELAAGVAYTVTVNDVSFNFGSENAGKTVTLGSANSNVKFT<br>GTNSDNQTETNVSTLKVKLDQNGVASLTNVSIANVYAINTTDNS
pore-forming complex	>SlpA<br>ATTINASSSAINTNTNAKYDVDVTPSVSAVAANTANNTPAIAGNLTGTISASYNGKTYTANL<br>KADTENATITAAGSTTAVKPAELAAGVAYTVTVNDVSFNFGSENAGKTVTLGSANSNVKFT<br>GTNSDNQTETNVSTLKVKLDQNGVASLTNVSIANVYAINTTDNSNVNFYDVTSGATVTNGA<br>VSVNADNQGQVNVANVVAAINSKYFAAQYADKKLNTRTANTEDAIKAALKDQKIDVNSVG<br>YFKAPHTFTVNVKATSNTNGKSATLPVVVTVPNVAEPTVASVSKRIMHNAYYYDKDAKRV<br>GTDSVKRYNSVSVLPNTTTINGKAYYQVVENGKAVDKYINAANIDGTKRTLKHNAYVYASS<br>KKRANKVVLKKGEVVTTYGASYTFKNGQKYYKIGDNTDKTYVKVANFR<br>>SlpA<br>ATTINASSSAINTNTNAKYDVDVTPSVSAVAANTANNTPAIAGNLTGTISASYNGKTYTANL<br>KADTENATITAAGSTTAVKPAELAAGVAYTVTVNDVSFNFGSENAGKTVTLGSANSNVKFT<br>GTNSDNQTETNVSTLKVKLDQNGVASLTNVSIANVYAINTTDNSNVNFYDVTSGATVTNGA<br>VSVNADNQGQVNVANVVAAINSKYFAAQYADKKLNTRTANTEDAIKAALKDQKIDVNSVG<br>YFKAPHTFTVNVKATSNTNGKSATLPVVVTVPNVAEPTVASVSKRIMHNAYYYDKDAKRV<br>GTDSVKRYNSVSVLPNTTTINGKAYYQVVENGKAVDKYINAANIDGTKRTLKHNAYVYASS<br>KKRANKVVLKKGEVVTTYGASYTFKNGQKYYKIGDNTDKTYVKVANFR<br>>SlpA2<br>NVNFYDVTSGATVTNGAVSVNADNQGQVNVANVVAAINSKYFAAQYADKKLNTRTANTE<br>DAIKAALKDQKIDVNSVGYFKAPHTFTVNVKATSNTNGKSATLPVVVTVPN<br>>SlpA2<br>NVNFYDVTSGATVTNGAVSVNADNQGQVNVANVVAAINSKYFAAQYADKKLNTRTANTE<br>DAIKAALKDQKIDVNSVGYFKAPHTFTVNVKATSNTNGKSATLPVVVTVPN

Table S7. Calculation of dissociation constants. K_d for SlpA_amy_III and GroP trimer by NMR according to formula in the 'Materials and Methods' section resulting in a mean K_d value of 3.5 mM.

GroP trimer

SlpA_amy_III residue	K_d [M]	K_d error	mean K_d	standard deviation
334	5.23E-03	1.17E-03	3.45E-03	6.24E-03
335	3.63E-03	5.26E-04		
336	4.92E-03	1.06E-03		
337	2.49E-03	5.99E-04		
338	3.86E-02	1.49E-02		
339	3.97E-02	4.65E-02		
340	3.11E-03	1.13E-03		
341	3.24E-03	5.13E-04		
342	9.02E-03	4.30E-03		
346	3.40E-03	1.92E-04		
348	1.09E-02	1.61E-03		
349	1.26E-02	4.31E-03		
350	5.26E-03	5.30E-04		
352	2.60E-03	6.51E-04		
353	2.39E-03	4.13E-04		
354	8.10E-03	2.06E-03		
355	8.56E-03	8.77E-04		
356	7.91E-03	7.91E-04		
357	1.20E-02	3.51E-03		
358	2.17E-03	3.95E-04		
362	2.36E-03	8.69E-04		
364	1.33E-02	1.06E-03		
365	4.37E-03	3.08E-04		
365	5.78E-03	3.79E-04		
366	1.03E-02	2.91E-03		
366	2.40E-03	3.33E-04		
367	4.67E-03	1.44E-03		
367	1.58E-02	5.11E-03		
368	2.83E-03	6.35E-04		
369	1.02E-02	1.78E-03		
370	8.89E-03	3.19E-03		
371	1.79E-03	1.51E-04		
374	7.55E-03	1.61E-03		
375	7.09E-03	1.03E-03		
376	3.07E-03	4.39E-04		
377	4.83E-03	1.37E-03		
378	3.85E-03	8.89E-04		
379	7.95E-03	2.24E-03		

380	3.66E-03	7.42E-04
381	9.34E-03	1.59E-03
382	6.45E-03	6.42E-04
383	2.17E-03	2.93E-04
384	6.18E-03	1.87E-03
385	3.44E-03	8.98E-04
386	1.80E-03	3.55E-04
387	2.43E-03	6.55E-04
388	1.11E-03	1.94E-04
389	5.86E-03	1.16E-03
390	8.45E-03	1.07E-03
391	7.98E-03	1.93E-03
393	6.14E-03	2.17E-03
394	5.68E-03	4.66E-04
396	4.44E-03	4.30E-04
397	7.48E-03	1.87E-03
398	3.81E-03	6.36E-04
399	2.89E-03	3.95E-04
400	2.82E-03	3.30E-04
401	1.78E-03	2.60E-04
402	4.32E-03	2.41E-04
409	9.88E-04	9.25E-05
410	9.49E-04	1.19E-04
413	3.89E-03	6.12E-04
414	2.17E-03	1.91E-04
415	2.46E-03	3.07E-04
416	7.10E-04	2.29E-05
425	1.16E-03	8.92E-05
426	8.81E-04	1.25E-04
427	2.95E-03	7.83E-04
428	2.72E-03	6.03E-04
429	5.17E-04	4.31E-05
431	8.48E-03	1.79E-03
438	1.10E-03	1.02E-04
439	1.18E-03	1.42E-04
440	7.54E-04	1.05E-04
441	1.05E-03	1.14E-04
442	2.06E-03	2.94E-04
444	5.47E-03	9.66E-04
445	1.79E-03	2.00E-04
446	1.30E-03	3.70E-04
449	1.06E-03	1.16E-04
450	3.46E-03	1.21E-03

454	7.17E-04	4.67E-05
455	6.33E-04	4.67E-05
456	5.61E-04	1.84E-05
457	2.26E-03	1.96E-04
458	1.77E-03	2.49E-04

Table S8. Calculation of dissociation constants. K_d for SlpA_amy_III and GroP pentamer by NMR according to formula in the ‘Materials and Methods’ section resulting in a mean K_d value of 0.5 mM.

GroP pentamer

SlpA_amy_III residue	Kd [M]	Kd error	mean Kd [M]	standard deviation
332	1.95E-03	5.26E-04	4.96E-04	4.79E-04
333	1.45E-03	2.98E-04		
334	1.60E-03	4.01E-04		
335	1.26E-03	3.75E-04		
336	5.56E-04	7.71E-05		
337	2.06E-03	5.11E-04		
349	4.08E-04	5.07E-05		
352	4.94E-04	1.31E-04		
353	3.84E-04	4.93E-05		
355	3.94E-04	2.08E-04		
356	2.17E-04	2.86E-05		
364	8.70E-04	2.71E-04		
366	8.45E-04	1.98E-04		
369	7.01E-04	1.53E-04		
370	6.64E-04	1.81E-04		
371	1.24E-03	2.28E-04		
376	2.42E-04	3.20E-05		
377	4.44E-04	3.18E-05		
378	4.19E-04	2.55E-05		
381	3.59E-04	4.06E-05		
382	3.49E-04	4.36E-05		
384	8.11E-04	1.13E-04		
386	6.24E-04	2.07E-04		
389	4.02E-04	8.91E-05		
390	3.17E-04	3.79E-05		
391	5.84E-04	6.25E-05		
410	4.97E-04	6.21E-05		
416	2.00E-04	1.49E-05		
425	2.34E-04	3.26E-05		
426	4.00E-04	1.48E-05		
438	3.40E-04	2.52E-05		
439	4.03E-04	1.22E-04		
441	1.53E-04	1.48E-05		
446	5.71E-04	1.19E-04		
449	9.31E-04	2.14E-04		
454	5.67E-04	7.40E-05		

Table S9. HADDOCK output scores of highest ranked clusters.

**Binding site 1**	
HADDOCK score	-39.5 +/- 4.3
Cluster size	18
RMSD from the overall lowest-energy structure	0.3 +/- 0.2
Van der Waals energy	-28.8 +/- 1.6
Electrostatic energy	-161.8 +/- 19.1
Desolvation energy	0.7 +/- 1.5
Restraints violation energy	47.5 +/- 30.8
Buried Surface Area	727.9 +/- 26.0
Z-Score	-1.6
**Binding site 2**	
HADDOCK score	-38.5 +/- 1.5
Cluster size	24
RMSD from the overall lowest-energy structure	0.3 +/- 0.1
Van der Waals energy	-24.2 +/- 0.6
Electrostatic energy	-128.3 +/- 13.7
Desolvation energy	-1.5 +/- 0.8
Restraints violation energy	0.4 +/- 0.5
Buried Surface Area	673.0 +/- 15.7
Z-Score	-2.0

Movie S1. Composition of SlpA layer model based on experimental crystal structures.
This movie shows how the proposed SlpA layer model comprises experimental crystal structures. The starting point is the experimental SlpA_ac_I crystal structure, which consists of coils of SlpA_I · SlpA_I dimers connected by their N-termini. The first part of the movie shows how these SlpA_I · SlpA_I dimer chains can be uncoiled and placed on a 2D plane. The movie's second part shows how the SlpA_I ·SlpA_I dimer chains are complemented by SlpA_II and SlpA_III domains (shown in 2 SpA_motifs) and how they assemble to the complete SlpA layer.

SI References

1. Smit, E., Jager, D., Martinez, B., Tielen, F. J. & Pouwels, P. H. Structural and functional analysis of the S-layer protein crystallisation domain of Lactobacillus acidophilus ATCC 4356: Evidence for protein-protein interaction of two subdomains. *J Mol Biol* **324**, 953–964 (2002).
2. Kay, L. E., Torchia, D. A. & Bax, A. Backbone dynamics of proteins as studied by nitrogen-15 inverse detected heteronuclear NMR spectroscopy: application to staphylococcal nuclease. *Biochemistry* **28**, 8972–8979 (1989).
3. Cavanagh, J., Fairbrother, W. J., Palmer, A. G., Rance, M. & Skelton, N. J. Relaxation and Dynamik Processes. in *Protein NMR Spectroscopy* 333–404 (Elsevier, 2007).
4. Jumper, J. *et al.* Highly accurate protein structure prediction with AlphaFold. *Nature* **596**, 583–589 (2021).
5. Evans, R. *et al.* Protein complex prediction with AlphaFold-Multimer. *bioRxiv* 2021.10.04.463034 (2022).
6. Smit, E., Oling, F., Demel, R., Martinez, B. & Pouwels, P. H. The S-layer protein of Lactobacillus acidophilus ATCC 4356: Identification and characterisation of domains responsible for S-protein assembly and cell wall binding. *J Mol Biol* **305**, 245–257 (2001).
7. Tang, G. *et al.* EMAN2: An extensible image processing suite for electron microscopy. *J Struct Biol* **157**, 38–46 (2007).
8. Pettersen, E. F. *et al.* UCSF ChimeraX: Structure visualization for researchers, educators, and developers. *Protein Science* **30**, 70–82 (2021).
9. Goddard, T. D. *et al.* UCSF ChimeraX: Meeting modern challenges in visualization and analysis. *Protein Science* **27**, 14–25 (2018).
10. Hendlich, M., Rippmann, F. & Barnickel, G. LIGSITE: automatic and efficient detection of potential small molecule-binding sites in proteins. *J Mol Graph Model* **15**, 359–363 (1997).
11. Steinkellner, G., Rader, R., Thallinger, G. G., Kratky, C. & Gruber, K. VASCo: computation and visualization of annotated protein surface contacts. *BMC Bioinformatics* **10**, 32 (2009).
12. Baranova, E. *et al.* SbsB structure and lattice reconstruction unveil Ca2+ triggered S-layer assembly. *Nature* **487**, 119–122 (2012).
13. Pavkov, T. *et al.* The Structure and Binding Behavior of the Bacterial Cell Surface Layer Protein SbsC. *Structure* **16**, 1226–1237 (2008).
14. Dordic, A. *et al.* Crystallization of domains involved in self-assembly of the S-layer protein SbsC. *Acta Crystallogr Sect F Struct Biol Cryst Commun* **68**, 1511–1514 (2012).
15. Pavkov, T. *et al.* Crystallization and preliminary structure determination of the C-terminal truncated domain of the S-layer protein SbsC. *Acta Cryst D* **59**, 1466–8 (2003).
16. Kroutil, M. *et al.* Towards the structure of the C-terminal part of the S-layer protein SbsC. *Acta Cryst F* **65**, 1042–1047 (2009).
17. Kern, J. *et al.* Structure of Surface Layer Homology (SLH) Domains from Bacillus anthracis Surface Array Protein. *J Bio Chem* **286**, 26042–26049 (2011).

18. Fioravanti, A. *et al.* Structure of S-layer protein Sap reveals a mechanism for therapeutic intervention in anthrax. *Nat Microbiol* **4**, 1805–1814 (2019).
19. Sychantha, D. *et al.* Molecular Basis for the Attachment of S-Layer Proteins to the Cell Wall of Bacillus anthracis. *Biochemistry* **57**, 1949–1953 (2018).
20. Sogues, A. *et al.* Structure and function of the EA1 surface layer of Bacillus anthracis. *Nat Commun* **14**, 7051 (2023).
21. Lanzoni-Mangutchi, P. *et al.* Structure and assembly of the S-layer in C. difficile. *Nature Communications 2022 13:1* **13**, 1–13 (2022).
22. Fagan, R. P. *et al.* Structural insights into the molecular organization of the S-layer from Clostridium difficile. *Mol. Microbiol.* **71**, 1308–1322 (2009).
23. von Kügelgen, A. *et al.* Interdigitated immunoglobulin arrays form the hyperstable surface layer of the extremophilic bacterium Deinococcus radiodurans. *Proc Natl Acad Sci U S A* **120**, e2215808120 (2023).
24. Herdman, M. *et al.* High-resolution mapping of metal ions reveals principles of surface layer assembly in Caulobacter crescentus cells. *Structure* **30**, 215-228.e5 (2022).
25. von Kügelgen, A. *et al.* In Situ Structure of an Intact Lipopolysaccharide-Bound Bacterial Surface Layer. *Cell* **180**, 348-358.e15 (2020).
26. Herrmann, J. *et al.* A bacterial surface layer protein exploits multistep crystallization for rapid self-assembly. *Proc Natl Acad Sci U S A* **117**, 388–394 (2020).
27. Bharat, T. A. M. *et al.* Structure of the hexagonal surface layer on Caulobacter crescentus cells. *Nat Microbiol* **2**, 17059 (2017).
28. von Kügelgen, A., Alva, V. & Bharat, T. A. M. Complete atomic structure of a native archaeal cell surface. *Cell Rep* **37**, 110052 (2021).
29. Jing, H., Takagi, J., Liu, J. & Springer, T. A. Archaeal Surface Layer Proteins Contain β Propeller, PKD, and β Helix Domains and Are Related to Metazoan Cell Surface Proteins. *Structure* **10**, 1453–1464 (2002).
30. Arbing, M. A. *et al.* Structure of the surface layer of the methanogenic archaean Methanosarcina acetivorans. *Proc Natl Acad Sci U S A* **109**, 11812–11817 (2012).
31. Gambelli, L. *et al.* Structure of the two-component S-layer of the archaeon Sulfolobus acidocaldarius. *Elife* **13**, (2024).
